# Differential abundance analysis of mesocarp protein from high- and low-yielding oil palms associates non-oil biosynthetic enzymes to lipid biosynthesis

**DOI:** 10.1186/s12953-015-0085-2

**Published:** 2015-11-26

**Authors:** Tony Eng Keong Ooi, Wan Chin Yeap, Leona Daniela Jeffery Daim, Boon Zean Ng, Fong Chin Lee, Ainul Masni Othman, David Ross Appleton, Fook Tim Chew, Harikrishna Kulaveerasingam

**Affiliations:** Sime Darby Technology Centre Sdn. Bhd., UPM-MTDC Technology Centre III, Lebuh Silikon, Universiti Putra Malaysia, 1st Floor, Block B, 43400 Serdang, Selangor Malaysia; Agro-Biotechnology Institute Malaysia, National Institutes of Biotechnology Malaysia, c/o MARDI Headquarters, 43400 Serdang, Selangor Malaysia; Department of Biological Sciences, Faculty of Science, National University of Singapore, Kent Ridge Road, Singapore, 117543 Singapore

**Keywords:** Oil palm, Elaeis guineensis, Yield, Lipid biosynthesis, Plant proteome

## Abstract

**Background:**

The oil palm *Elaeis guineensis* Jacq. which produces the highest yield per unit land area of the oil crops is the most important commercial oil crop in South East Asia. The fleshy mesocarp of oil palm fruit, where oil is mostly derived from, contains up to 90 % dry weight of oil (one of the most concentrated in plant tissues). Hence, there is attention given to gain insights into the processes of oil deposition in this oil rich tissue. For that purpose, two-dimensional differential gel electrophoresis (DIGE) coupled with western assays, were used here to analyze differential protein levels in genetically-related high-and low-yielding oil palm mesocarps.

**Results:**

From the DIGE comparative analysis in combination with western analysis, 41 unique differentially accumulated proteins were discovered. Functional categorization of these proteins placed them in the metabolisms of lipid, carbohydrate, amino acids, energy, structural proteins, as well as in other functions. In particular, higher abundance of fructose-1,6-biphosphate aldolase combined with reduced level of triosephosphate isomerase and glyceraldehyde-3-phosphate dehydrogenase may be indicative of important flux balance changes in glycolysis, while amino acid metabolism also appeared to be closely linked with oil yield.

**Conclusions:**

Forty-one proteins in several important biological pathways were identified as exhibiting differential in abundance at critical oil production stages. These confirm that oil yield is a complex trait involving the regulation of genes in multiple biological pathways. The results also provide insights into key control points of lipid biosynthesis in oil palm and can assist in the development of genetic markers for use in oil palm breeding programmes.

**Electronic supplementary material:**

The online version of this article (doi:10.1186/s12953-015-0085-2) contains supplementary material, which is available to authorized users.

## Background

The oil palm *Elaeis guineensis* Jacq. produces the highest yield per unit land area of the oil crops [1] and has become the most important commercial oil crop in tropical South East Asia. The oil palm fruit is a drupe with bunches containing 1000–3000 fruitlets and most of the commercially extracted oil is derived from its fleshly mesocarp. The mesocarp contains up to 90 % dry weight of oil, one of the most concentrated found in plant tissues [2, 3]. Yield remains the main focus in oil palm cultivation due to the limitation of available arable land and growing demand for edible and non-edible oils. Current standard commercial oil palm material can produce approximately 4 ton/hectare/year of oil at a planting density of 148 palms/hectare. However, some plants have attained yields greater than 10 ton/hectare/year in trial plots giving an indication of potential future yields.

Recent advances in biochemical analysis have allowed researchers to develop omics techniques to investigate crop traits at a cellular level. In particular, proteomics has been used extensively to investigate the biochemical processes attribute to traits in non-oil crops [4–9], as well as processes leading to increased lipid production in oil crops [10–15]. The proteomes of two near-isogenic sunflower varieties differing in seed oil traits were comparatively analyzed by Hajduch et al. [10]. Proteins associated with glycolytic pathway were found highly up-regulated in the high oil variety. In contrast, proteins associated to amino acid biosynthesis were up-regulated in the low oil variety. Another group of researchers, Troncoso-Ponce et al. [11] investigated the glycolytic initial metabolites and enzyme activities from developing seed of two different sunflower lines, of high and low oil content, during storage lipid accumulation. They discovered that the amount of sucrose produced and available for lipid synthesis was higher in high oil line. In addition, the enzymatic activities of both phosphoglycerate kinase and enolase were found notably higher in this line, allowing increased synthesis of phosphoenolpyruvate, an intermediate carbon donor for lipid biosynthesis. Another comparative analysis of the soluble proteins found in early developing germs from high-oil and normal inbred lines in maize [12], revealed that 3 enzymes closely related to lipid biosynthesis, namely enoyl-ACP reductase, stearoyl-ACP desaturase and acetyl-CoA C-acyl-transferase, had higher abundance in high-oil lines than in normal.

Recently, several studies that employed comparative transcriptomics and metabolomics to gain insight into the development of oil palm fruit have been reported [16–21]. Bourgis et al. [16] examined the differences in transcriptome and metabolome between oil palm and date palm during mesocarp development, in the hope to elucidate the mechanisms that lead to an extreme difference in carbon partitioning in them (the mesocarp of oil palm accumulates oil while the mesocarp of date palm accumulates sugars). They implicated that synthesis of fatty acids and supply of pyruvate in the plastid, rather than acyl assembly into triacylglcerol, as the main factor for the accumulation of oil in the mesocarp of oil palm. Tranbarger et al. [17] and Dussert et al. [21] investigated the transcriptional basis of lipid accumulation in the mesocarp of oil palm. A transcript, homologous to *Arabidopsis* seed oil transcription factor WRINKLED1, was identified to coordinate its transcript level with several fatty acids biosynthetic transcripts and high rates of lipid deposition, suggesting the mesocarp homolog is an important regulatory factor in oil biosynthesis.

The DIGE (Difference Gel Electrophoresis) analysis [22, 23], a two dimensional gel electrophoresis employing sensitive fluorescent labeling dyes, has been successfully employed in protein expression analyses in rice and sunflower [10, 24]. Although DIGE has been utilized in the studies of quantitative proteomics in these plants, but none in oil palm. In this study, we report the first comparative proteomics analysis of two distinct groups of oil palms (low-yielding, LY palms and high-yielding, HY palms) using DIGE analysis, followed by confirmation using western blot immunoassays, to identify proteins associated with yield in oil palm and provide insights into the regulation of lipid biosynthesis.

## Results and discussion

### Mesocarp tissue extraction protocol for 2D gel electrophoresis

**Fig. 1:**
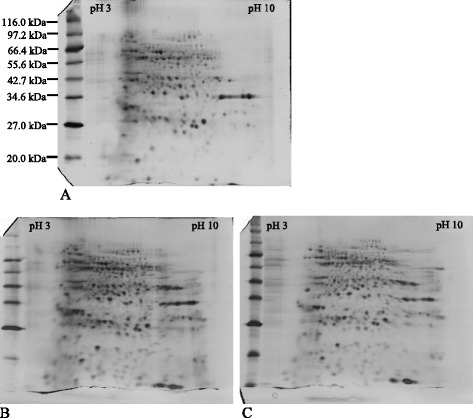
Evaluation of extraction protocols for 2D gels. Three methods were compared. Silver-stained gels were produced after running IEF for 9 kVh using 7 cm IEF strips of pH3-10NL, with similar H16WAP samples (3 μg/gel). Gel B and C are better 2D gels compared to A. Both are comparable. [**a**: original sucrose method; **b**: modified sucrose method; **c**: phenol/ammonium acetate method]

Three methods were tested for protein extraction from oil palm mesocarp tissues. They were (i) the sucrose method published by He et al. [25] for pine needles, (ii) a modified sucrose method and (iii) the phenol/ammonium acetate method [26]. The sucrose method was modified in this study to give a better 2D gels, evidently with more protein spots being resolved in the alkaline range (Fig. 1). Moreover, the mean number of protein spots detected in 2D gels (3 replicate gels) produced from the original sucrose method was 576 ± 18 spots whilst 738 ± 80 spots detected in 2D gels from the modified sucrose method. Successively, the modified sucrose method was compared to the phenol/ammonium acetate method. In terms of spot resolution and separation, the 2D gels produced from these 2 methods were comparable (Fig. 1) in that 729 ± 78 spots were detected from 2D gels of the third method. Consequently, the modified sucrose method was adopted to extract protein from oil palm mesocarp tissues for subsequent 2D gel electrophoresis study. Moreover, the latter method involves the usage of toxic phenol and procedurally lengthy.

### Selection of plant materials and comparative studies

**Fig. 2:**
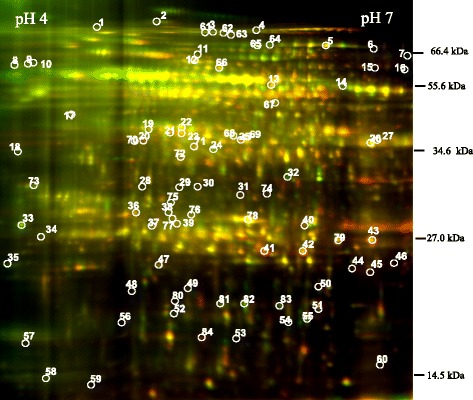
Overlay of Cy3 and Cy5 gel images of a 2-dimensional DIGE gel. DIGE gels were produced for spot analysis using 13 cm IEF strips of pH 4–7 with 20-kVh focusing. Mesocarp protein from high yielding palms was labeled with Cy3 whilst mesocarp protein from low yielding palms with Cy5. A total of nine DIGE gels were produced for analysis from three biological replicates of 12, 16 and 18 WAP fruit. Numbers in the gel indicate the geographical position of protein spots in the map

**Fig. 3:**
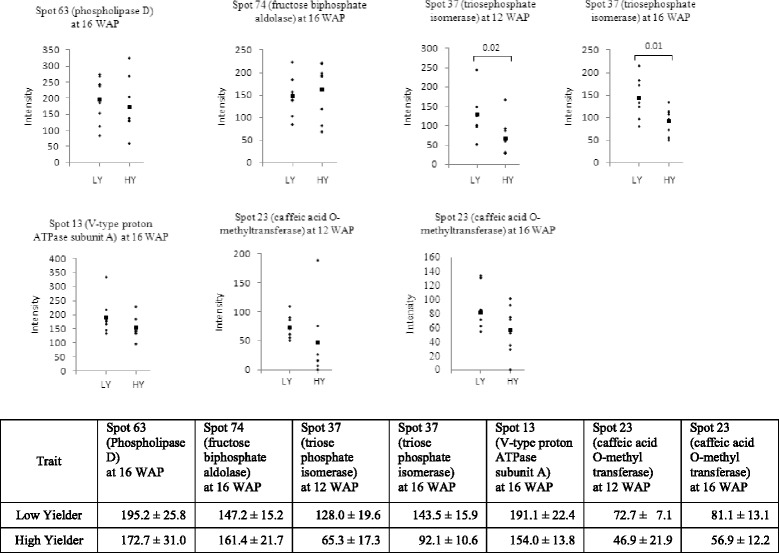
Enzyme levels in LY and HY palm mesocarp tissues (*n* = 8). Intensity data were generated from dot blot immunoassay using antibodies against the respective enzymes. (■)indicates the mean of each group. Significant Student’s *t*-test *p*-values are shown. The mean and standard error are given in the accompanying table

**Fig. 4:**
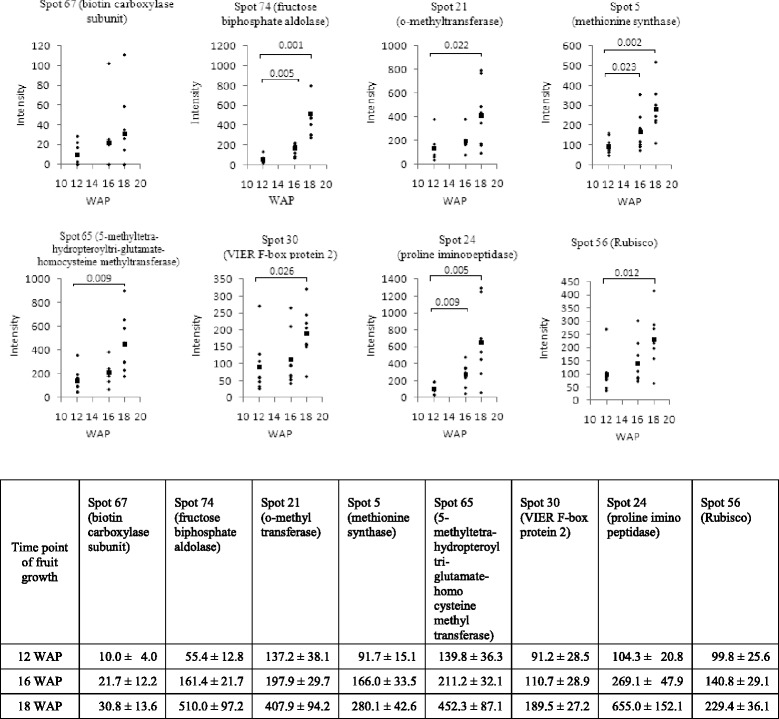
Temporal level of differential proteins found in HY palm mesocarp tissue during fruit development at 12–18 WAP (*n* = 8). Intensity data were generated from dot blot immunoassay using antibodies against the respective enzymes. (■) indicates the mean of each group. Significant Student’s *t*-test p-values are shown. The mean and standard error are depicted in the accompanying table

In order to investigate biosynthetic controls leading to significant increases in yield, the proteomes of genetically related oil palms grown in the same field and exhibiting a 2-fold difference in oil yield [19] were selected for this study. Mesocarp samples were collected at three key phases preceding and during lipid biosynthesis: 12 weeks after pollination (WAP) (preceding), 16 WAP (onset of lipid deposition) and 18 WAP [3, 17, 27–29]. The DIGE protein analysis was used to identify pairwise differential proteins between three high-yielding (HY) and three comparatively low-yielding (LY) plants. A total of nine analytical gels were produced (Fig. 2; see all DIGE gels in Additional file 1) from the eighteen samples and subsequently used for cross-gel Biological Variation Analysis (BVA) (DeCyder™ 2D software Version 6.5 by Amersham BioSciences): HY versus LY at 12, 16 and 18 WAP, in addition to temporal analysis of HY palms: 16 and 18 WAP versus 12 WAP (Additional file 2). Out of approximately 2000 peptide spots detected, 84 were found to be at least 1.5-fold different and have a *p*-value < 0.1. The *p*-value < 0.1 was taken to compensate the small sample size (only 3 biological replicates) in this DIGE experiment. Moreover, the DIGE experiment here functioned mainly as a screening tool for subsequent western analysis that involved bigger populations. Mass spectrometry identification using MALDI-TOF/TOF was conducted on 61 distinct spots with isoforms removed. Fifty-three protein spots were identified based on combined MS and MS/MS matching to proteins in the public domain of NCBI non-redundant database, of which 45 were unique (see Additional file 3 for MASCOT protein-peptide identification results). Subsequently, the DIGE results were cross-checked with western analysis by using a larger sample size of 8 biological replicates (Figs. 3 and 4, Additional file 4, Additional file 5, Additional file 6, Additional file 7).

The functions of the majority of the differential proteins of those having significant *p*-value < 0.05 from DIGE and western analyses, a total of 41 proteins, could be grouped into six main categories (Table 1) according to the Kyoto Encyclopedia of Genes and Genomes (KEGG) pathways database [30]: lipid metabolism, carbohydrate metabolism, amino acid metabolism, energy metabolism, stress-related proteins and structure-related proteins. Oil yield is known to be a complex trait involving the regulation of many genes across multiple biological pathways [31]. In this study, only 2 of the 41 proteins are known to be involved in lipid biosynthesis, while the remaining are involved in a range of biosynthetic pathways.

**Table 1:** Unique proteins expressed differentially in HY versus LY palms at 12–18 WAP, or exhibiting temporal expression differences in HY palms

Spot #	Protein name^a^	NCBI Accession #	Protein MW	Protein PI	Pep.Count	Protein score	Protein Score C. I. %	Total ion score	Total Ion C. I. %	Expression difference HY versus LY (WAP)^b^		Temporal expression trend in HY (WAP)^b^	
										DIGE	Western analysis	DIGE	Western analysis
	Lipid metabolism												
63	phospholipase D [*Oryza sativa*]	gi|1020415	92151	5.6	11	130	100.0	40	67.4	down (16)	down (16)	down (16)	up (16, 18)
67	biotin carboxylase subunit [*Glycine max*]	gi|3219361	58770	7.2	11	302	100.0	222	100.0	-	-	up (16, 18)	up (16, 18)
	Carbohydrate metabolism												
26	glyceraldehyde 3-phosphate dehydrogenase [*Elaeis guineensis*]	gi|82400215	32136	7.1	7	191	100.0	122	100.0	down (16)	down (12, 16)	-	up (18)
74	fructose-biphosphate aldolase [*Elaeis guineensis*]	gi|192910908	38578	6.7	8	174	100.0	119	100.0	up (16)	up (16)	up (16, 18)	up (16, 18)
37	Triosephosphate isomerase, chloroplastic [*Secale cereale*]	gi|1174745	31613	6.0	7	103	100.0	56	99.4	down (16)	down (12, 16)	-	up (18)
	Energy metabolism												
44	NADPH:quinone reductase family protein [*Arabidopsis thaliana*]	gi|30687535	21778	6.1	4	185	100.0	155	100.0	down (12)	nt	up (16)	nt
46	FQR1 (flavodoxin-like quinone reductase 1) [*Arabidopsis thaliana*]	gi|15239652	21782	6.0	4	130	100.0	100	100.0	down (12)	nt	up (16)	nt
75	short chain type dehydrogenase [*Ricinus communis*]	gi|223550344	33332	9.5	3	104	100.0	88	100.0	-	nt	up (16, 18)	nt
51	mitochondrial peroxiredoxin [*Pisum sativum*]	gi|47775654	21463	8.4	3	140	100.0	120	100.0	up (16)	down (16)	up (16, 18)	up (18)
	Amino acid metabolism												
21	o-methyltransferase [*Ricinus communis*]	gi|223540510	40000	5.3	4	92	100.0	68	99.9	down (16)	down (16)	down (16, 18)	up (18)
5	methionine synthase protein [*Sorghum bicolor*]	gi|18483235	83736	5.9	12	211	100.0	166	100.0	down (16)	-	-	up (16, 18)
65	5-methyltetrahydropteroyltri-glutamate-homocysteine methyltransferase [*Oryza sativa*]	gi|108862992	84582	5.9	9	84	99.7	39	71.7	-	-	down (16, 18)	up (18)
30	VFB2 (VIER F-BOX PROTEINE 2); ubiquitin-protein ligase [*Arabidopsis thaliana*]	gi|18409012	57212	8.7	8	65	80.5	-	-	up (18)	down (18)	-	up (18)
24	proline iminopeptidase, putative [*Ricinus communis*]	gi|223538638	44547	6.0	5	94	100.0	71	100.0	up (18)	up (18)	up (16, 18)	up (16, 18)
	Stress related proteins												
80	17.6 kDa class I small heat shock protein (HSP17.6C-CI) (AA 1–156) [*Arabidopsis thaliana*]	gi|15220832	17593.0	5.4	5	70	94.0	30	-	-	-	up (16. 18)	up (18)
7	OSJNBb0085F13.17 [*Oryza sativa*]	gi|38345312	80199.8	5.1	12	102	100.0	28	-	down (16)	nt	-	nt
54	glutathione peroxidase [*Triticum monococcum*]	gi|148529480	11668.8	5.7	3	73	97.0	50	97.9	up (16)	down (16)	-	up (18)
41	glutathione S-transferase Phi [*Matricaria chamomilla*]	gi|17385642	25264.9	6.0	5	96	100.0	69	100.0	down (16)	-	up (16)	up (16, 18)
43	glutathione-S-transferase theta, gst, putative [*Ricinus communis*]	gi|223528475	24482.6	5.8	1	82	99.6	77	100.0	up (18)	nt	up (16, 18)	nt
78	abscisic stress ripening protein [*Musa acuminata*]	gi|219963178	16735.8	5.6	3	76	98.5	51	97.8	-	nt	down (16, 18)	nt
53	temperature-induced lipocalin [*Elaeis guineensis*]	gi|192911934	21614.8	5.7	5	93	100.0	51	98.8	up (16)	-	up (16)	up (18)
	Structure related proteins												
22	actin [*Populus trichocarpa*]	gi|224117708	40592.4	5.1	15	375	100.0	258	100.0	down (16)	-	-	up (18)
23	caffeic acid O-methyltransferase [*Vanilla planifolia*]	gi|45444737	40632.5	5.7	4	92	100.0	72	100.0	down (12, 16)	down (12, 16)	down (16, 18)	-
73	fibrillin-like protein [*Elaeis guineensis*]	gi|32250939	18991.0	5.0	7	109	100.0	37	46.0	up (12)	nt	down (16)	nt
68	Os12g0163700 (actine gamma 2) [*Oryza sativa*]	gi|115487492	39837.0	5.3	10	137	100.0	62	99.9	-	-	up (16, 18)	up (18)
	Other Proteins												
13	V-type proton ATPase catalytic subunit A [*Daucus carota*]	gi|137460	68792	5.3	17	250	100.0	107	100.0	down (16)	down (16)	down (16)	up (18)
9	catalase 2 [*Elaeis guineensis*]	gi|192910916	56922.4	6.5	11	100	100.0	25	-	up (18)	up (18)	-	up (18)
56	large subunit of ribulose-1, 5-bisphosphate carboxylase/oxygenase [*Chlorogonium elongatum*]	gi|74267421	41910.0	7.0	5	71	95.3	40	73.0	up (18)	up (18)	up (16, 18)	up (18)
32	Ran GTPase binding protein [*Ricinus communis*]	gi|223434074	119713.4	9.0	12	67	87.9	-	-	up (12)	-	up (16, 18)	up (18)
72	ribosomal protein L10 [*Elaeis guineensis*]	gi|192910684	34609.4	5.4	6	251	100.0	198	100.0	down (12)	down (12)	up (16, 18)	up (16, 18)
49	ABC1 family protein [*Arabidopsis thaliana*]	gi|79325958	73790.7	5.6	13	73	96.7	-	-	down (18)	-	down (18)	up (16, 18)
48	Lea1P [*Daucus carota*]	gi|10945667	16361.9	7.8	7	66	84.5	-	-	up (16)	-	up (16)	up (18)
33	nascent polypeptide associated complex alpha [*Vitis vinifera*]	gi|225470846	22013.8	4.3	5	305	100.0	262	100.0	up (12)	down (12)	down (16, 18)	up (16, 18)
58	Os02g0753300 [*Oryza sativa*]	gi|115448731	19972.8	6.2	2	87	99.9	71	100.0	up (16)	nt	-	nt
66	Os05g0482700 [*Oryza sativa*]	gi|115464537	60774.8	5.3	10	256	100.0	183	100.0	-	nt	down (16)	nt
84	regulator of ribonuclease activity A [*Zea mays*]	gi|195638112	18105.2	5.8	5	80	99.4	34	-	-	nt	up (16, 18)	nt
36	retroelement pol polyprotein-like [*Arabidopsis thaliana*]	gi|9759493	126284.1	9.0	15	66	85.2	-	-	down (12)	nt	up (16)	nt
59	conserved hypothetical protein [*Ricinus communis*]	gi|223539131	16333.4	6.6	1	55	-	49	97.5	up (16)	nt	up (16, 18)	nt
34	predicted protein [*Ostreococcus lucimarinus* CCE9901]	gi|145345862	14575.0	4.6	3	66	85.2	46	92.4	up (16)	nt	up (16)	nt
79	predicted protein [*Physcomitrella patens*]	gi|168027637	23578.3	6.9	2	70	93.8	58	99.7	-	nt	up (16)	nt
55	hypothetical protein isoform 1 [*Vitis vinifera*]	gi|225434277	22608.8	8.7	5	88	99.9	54	99.0	up (12)	nt	down (18)	nt

^a^Identification of proteins was carried out using MASCOT against the NCBI non-redundant database

^b^Western dot blot immunoassay was conducted for validation. up = up regulated, down = down regulated, − = no differential, *nt* not tested

### Lipid metabolism

None of the key enzymes known to be directly involved in lipid biosynthesis or assembly was found to be differential between the HY and LY oil palm samples in this study, for example plastid fatty acid synthase and thioesterase, cytosolic acyl-CoA synthetase and the endoplasmic reticulum located enzymes of the Kennedy pathway [12, 32]. This could possibly due to the fact that the DIGE experiment here was limited to the detection of proteins within pH 4 to 7 only. The pH 4–7 range was chosen in this study because it is known that the majority of cellular proteins are found in this particular pH range. The pI of oil palm fatty acid synthases and thioesterases, and acyl-CoA synthetases, as well as the endoplastic reticulum located enzymes of the Kennedy pathway are not known. However, several *Arabidopsis* fatty acid synthases, fatty acid thioesterases and acyl-CoA synthetases were reported to have pI in the range above 7 [33–38]. Incidentally, the pI of some of the enzymes in the Kennedy pathway, viz the glycerol-3-phosphate *O*-acyltransferase, lysophosphatidic acid acyltransferase and diacylglycerol acyltransferase are reported to be in the alkaline range, respectively, pI 9.36 in flowering plant *Echium pitardii* [39], pI 9.43 in peanut [40] and pI 9.46 in microalga *Myrmecia incisa* [41].

Interestingly, the accumulation of phospholipase D at 16 WAP was observed to be significantly higher [1.6x, *p*-value < 0.05](Additional file 2) in LY palms compared to HY palms using DIGE (Table 1) and western analysis (Fig. 3). Phospholipase D cleaves the ester bond between the phosphatidyl moiety and the head group of phospholipids, but can also act as a transferase [42–44]. A study by Yu [45] found *Arabidopsis* phospholipase D knockout mutants exhibited decreased seed oil content compared to wild type, although it is possible that a reduction in the activity of this lipase at the period of highest lipid biosynthesis may be beneficial for yield.

Biotin carboxylase subunit was found to be up-regulated in the HY palms during lipid biosynthesis from 1.8-fold at 16 WAP to 3.0-fold at 18 WAP [*p*-value < 0.05] (Additional file 2) compared to 12 WAP when lipid biosynthesis is still relatively minimal (Fig. 4). Tranbarger et al. [17] also observed a higher abundance of biotin carboxylase transcripts during oil biosynthesis. Biotin carboxylase subunit is a component of acetyl-coenzyme A carboxylase [46]. Acetyl-CoA carboxylase catalyzes the conversion of acetyl-CoA to malonyl-CoA as the initial step of *de novo* fatty acid synthesis occurring in the plastid [46]. It has been demonstrated that over-expression of foxtail millet acetyl-CoA carboxylase gene in maize increases seed oil content by 25 to 65 % [47]. Although, no differential abundance was observed between HY and LY palms in this study, the importance of biotin carboxylase to lipid production in oil palm mesocarp is clearly evident.

### Carbohydrate metabolism

DIGE results identified three enzymes involved in glycolysis as being produced at a different levels between HY and LY palms; fructose-1,6-biphosphate aldolase (FBA), triosephosphate isomerase (TPI) and glyceraldehyde-3-phosphate dehydrogenase (GAPDH). Results of western analysis for FBA and TPI were concordant with DIGE data (Fig. 3). During the last stages of glycolysis, FBA fragments fructose-1,6-biphosphate into glyceraldehyde-3-phosphate (GAP) and dihydroxyacetonephosphate (DHAP), while TPI acts as a housekeeping enzyme to catalyze the inter-conversion and maintain appropriate balance of GAP and DHAP concentrations. Subsequently, GAP is converted to 1,3-bisphosphoglycerate by GAPDH and then eventually leads to pyruvate. Meanwhile, glycerol-3-phosphate dehydrogenase converts DHAP to glycerol 3-phosphate, an important intermediate of the acylglycerol backbone. FBA was observed to be up-regulated at 16 and 18 WAP, concordant with lipid biosynthesis (Fig. 4), and more interestingly have higher abundance in HY palms at 16 WAP (Fig. 3). The up-regulation of FBA in HY palms is likely to contribute to increased flux through glycolysis into *de novo* fatty acid synthesis [10, 48]. Interestingly, DIGE analysis indicated that TPI and GAPDH were both down-regulated in HY palms at 16 WAP. These findings are consistent with observations in sunflower where TPI and GAPDH activities were found to be higher in the low-oil content sunflower lines [11]. However, they also reported that FBA exhibited higher activity in low-oil lines. Studies of yeast [49] and canola [14] also demonstrated that reduced TPI activity causes a shift in glycolytic flux towards glycerol leading to increased yield. It is likely that a concurrent reduction in GAPDH activity would further alter the flux balance at this important branch point in the glycolysis pathway. Detailed metabolite comparisons of HY and LY oil palms provide further evidence of this flux balance change where glycerol-3-phosphate levels were observed to be 43 % higher in HY mesocarp at 16 WAP, while downstream of GAP, 3-phosphoglyceric acid levels were 26 % lower [19].

**Fig. 5:**
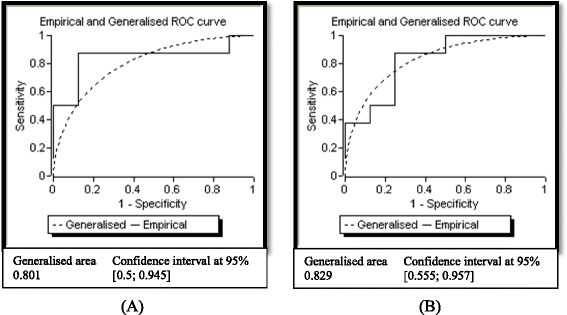
Receiver operating characteristic (ROC) curves plotted for triosephosphate isomerase enzyme at 12 (**a**) and 16 (**b**) WAP. Area under the curve greater than 0.75 is indicative of a good diagnostic marker

Receiver operating characteristic (ROC) curves [50] were generated using TPI protein levels detected in HY and LY palms at 12 and 16 WAP (Figs. 5). The generalized area under the curve for both time points was calculated to be 0.80 and 0.85, respectively, indicating the potential use of TPI as a yield biomarker.

### Amino acid metabolism

Several enzymes involved in amino acid metabolism were found to have significantly higher levels during lipid biosynthesis (Fig. 4). Although, no significant differences were observed in their levels between HY and LY palms. A separate study by Teh et al. [19] has shown there are significantly higher concentrations of amino acids in the mesocarp of HY oil palms at the onset of lipid biosynthesis. Tranbarger et al. [17] has also reported higher amino acid concentrations in oil palm compared to its non-oil-producing relative, date palm. Taken together, these results suggest that the regulation and metabolism of amino acids before and during lipid biosynthesis may play a crucial role in fruit maturation and oil yield.

### Structural protein

Our DIGE and western immunoassay results indicated that the enzyme caffeic acid *O*-methyltransferase (COMT) was more abundance in LY palms compared to HY palms at 12 WAP [2.1x, *p* < 0.05] and 16 WAP [3.3x, *p* < 0.05] (Table 1, Fig. 3 and Additional file 2). COMT is a key enzyme in the lignin biosynthetic pathway [51] and it is plausible that a reduction in lignin biosynthesis may be complimentary to higher lipid production in mesocarp tissue.

### Other proteins

Abundance of several other proteins were found to be altered in the course of oil palm fruit development. One is the Vacuolar H + −ATPase (V-ATPase). It is important for secondary ion transport into the expanding vacuole and as a regulator of membrane trafficking in higher plants [52]. In pear trees, V-ATPase activity is reported to be down-regulated during the flowering stage and markedly increases during early developmental stages and fruit growth [53, 54]. In oil palm mesocarp, the level of V-ATPase was observed to be higher in LY palms [1.9x, p < 0.05] (Additional file 2) at 16 WAP compared to HY palms using DIGE (Table 1) and western analysis (Fig. 3). The direct role of V-ATPase in contributing to the onset of oil biosynthesis in oil palm at 16 WAP is unknown. Our results here, however, suggested that this enzyme may associate with oil biosynthesis in oil palm.

Other proteins that showed differential abundance in HY palms have been reported to be associated with oil yield. Our DIGE analysis led to the identification of seven stress related proteins such as 17.6 kDa class I small heat shock protein [55], glutathione S-transferase [56] and glutathione peroxidase [57]. Studies of maize and sunflower have also found higher level of stress response proteins in high-yielding lines [10, 12]. We also observed that catalase was higher in abundance in the mesocarp of HY palms at 18 WAP (Additional file 2). Catalase plays a role in the regulation of hydrogen peroxide and reactive oxygen species, and has been reported to be important during sunflower and maize seed development and oil biosynthesis [13, 58, 59] as well as in peach mesocarp development [60]. Large subunit ribulose-1,5-bisphosphate caboxylase/oxygenase (Rubisco) is another protein found to have higher levels in HY palms at 18 WAP (Additional file 2). The abundance of Rubisco was also clearly correlated with lipid biosynthesis, increasing steadily from 12 to 18 WAP (Fig. 4). Rubisco is the most abundant protein found in plants and is known to play a crucial role in photosynthetic carbon assimilation. Studies on developing embryos of *Brassica napus* indicated that Rubisco increases the efficiency of carbon use for triacylglycerol production without the Calvin cycle [14, 61] and its expression has been linked with yield in maize [62, 63].

Several transcriptomics studies report the importance of transcription factor WRINKLED1 in oil biosynthesis in *Arabidopsis*, maize and oil palm [17, 18, 21, 64–67]. In oil palm mesocarp, Tranbarger et al. [17], Dussert et al. [21] and Wong et al. [18] all noted positive correlation between oil deposition during fruit maturity and *WRINKLED1* expression; mRNA of *WRINKLED1* increased from 12 WAP to 16 WAP and subsequently decreased from 18 WAP onwards. Conversely, the temporal changes of WRINKLED1 protein abundance during fruit development was not observed in the present DIGE study. Incidentally, alteration in protein level of any transcription factors was neither observed in DIGE analysis of maize differing in oil trait by Hadjuch et al. [10], nor in 2D gel comparative analyses of *Brassica campestri* oilseed development [68] or of *Olea europaea* olive fruit development [15]. Transcription factors are generally expressed in very low amount in a cell, both as RNA and protein, because only small quantity are required for activation of cognate genes. Hence, it is not impossible that the mesocarp WRINKLED1 protein is being produced in very low concentration that it is beyond the detection limit of the DIGE technique deployed here. In addition, it is not unknown that proteins of low abundance are being masked by proteins of higher abundance in this gel-based proteomics analysis [69, 70], thus making their detection challenging.

## Conclusions

In summary, 2D-DIGE techniques identified forty-one protein candidates as being differentially accumulated in two genetically related populations of oil palm displaying a 2-fold difference in yield. While only two of the proteins are known to be involved in oil metabolism, many are involved in other important pathways such as glycolysis, energy metabolism, amino acid metabolism and structural proteins. These results confirm that oil yield is a complex trait involving the regulation of genes in multiple biological pathways. In particular, two important glycolytic enzymes FBA and TPI exhibit differential levels in high-yielding palms, and suggest altered flux balance between DHAP and GAP may lead to increased yield. Results also showed that the regulation of amino acid metabolism appears to be closely linked to lipid biosynthesis. Better understanding of the biochemical differences leading to increased oil yield in *Elaeis guineensis* will aid the identification of key biosynthetic control points in addition to enabling the identification of markers for use in breeding programmes.

## Methods

### Plant material

Sixteen sibling or half-sibling palms derived from crosses of Serdang Avenue *dura* and AVROS *pisifera* and grown in the same field at Carey Island Estate, Malaysia were selected for this study. Oil yield and fruit bunch analysis data were collected for individual plants over a 7 year period. Eight palms were identified as high-yielding with yields of 10–12 t/Ha/y, and 8 were relatively low-yielding with 4–7 t/Ha/y [19]. Fruit bunches were harvested at 12, 16, and 18 weeks after pollination to represent key lipid biosynthesis phases [3, 17, 27–29]. Mesocarp tissues were collected, immediately frozen in liquid nitrogen then stored at−80 °C prior to use. The permission for sample collection was obtained from the owner of the estate, Sime Darby Plantation Malaysia. No specific permissions were required for the location and the field studies, because the project did not involve any protected or endangered plant species.

### Mesocarp protein extractions for 2D gel electrophoresis

In order to find a suitable protein extraction method for 2D gel experiments using mesocarp protein, 3 methods were compared. They were (i) the sucrose method [25], (ii) a modified sucrose method and (iii) the phenol/ammonium acetate method [26].

### The sucrose method

He et al [25] 10 mL sucrose extraction buffer (5 % sucrose, 4 % SDS and 5 % β-mercaptoethanol) and 0.1 g PVPP were added to 1 g pulverized tissue and the mixture was swirled gently for 10 min at room temperature. After centrifugation at 10,000 g for 5 min, the supernatant was heated at 100 °C for 3 min and cooled to room temperature. Eight volumes of cold acetone were then added to the cooled supernatant. The mixture was incubated at−20 °C for at least 1 h, prior to centrifugation at 10,000 g for 10 min. The resulting pellet was re-suspended in 5 mL sucrose extraction buffer before being centrifuged at 10,000 g for 10 min. The protein pellet was washed twice in 80 % cold acetone and finally precipitated in 4 volumes of cold acetone before air drying.

### The modified sucrose method

In the sucrose modified method, the steps found in the sucrose method [25] were followed from the start until the re-suspension of protein pellet in 5 mL sucrose extraction buffer. From here, the insoluble material was removed by centrifugation at 10,000 g for 5 min at room temperature. Sequentially, 4 volumes of cold acetone were added to the clear supernatant prior to incubation for 1 h at−20 °C. Following centrifuging for 10 min, the new pellet was washed twice in 80 % cold acetone and once in cold acetone before air drying. All centrifugal procedures in this method were carried out at 4 °C, unless stated otherwise.

### The phenol/ammonium acetate method

Two mL cold 10 % TCA/acetone was added to 300 mg of pulverized mesocarp tissue, and the mixture was thoroughly mixed before centrifuging at 16,000 g for 3 min at 4 °C. The supernatant was discarded and 2 mL ammonium acetate (0.1 M) in 80 % methanol was added to the pellet and the mix was vortexed prior to being centrifuged at 16,000 g for 3 min at 4 °C. The supernatant was discarded and the pellet was washed in 80 % acetone by vortexing. The mixture was centrifuged at 16,000 g for 3 min at 4 °C and the supernatant removed. The resulting pellet was air-dried at room temperature or incubated at least 50 °C to remove residual methanol. Sequentially, the protein in the dried pellet was extracted by vortexing vigorously in 2 mL of 1:1 phenol pH 8.0/SDS buffer [SDS buffer: 0.1 M Tris–HCl (pH 8.0), 30 % sucrose, 2 % SDS, 5 % β-mercaptoethanol] for 5 min. The mixture was centrifuged at 16,000 g for 3 min at 4 °C and the phenol phase (top phase) was collected. The protein in the phenol phase was precipitated by adding 2 mL methanol containing 0.1 M ammonium acetate and stored overnight at−20 °C. The protein was pelleted by centrifuging at 16,000 g for 5 min at 4 °C before being washed in methanol by vortexing. After centrifuged at 16,000 g for 3 min at 4 °C, the pellet was washed in 80 % acetone. A final centrifugation at 16,000 x g for 3 min at 4 °C was carried out and the supernatant discarded. The final pellet was allowed to air dry.

The protein pellet obtained from each method aforementioned was then re-suspended in lysis buffer containing 30 mM Tris–HCl (pH 8.8), 7 M urea, 2 M thiourea and 4 % CHAPS. The mixture was sonicated for 30 min at room temperature and centrifuged at 14,000 rpm for 30 min at room temperature and the resulting supernatant was collected. Protein concentration of the supernatant was determined using the Bradford assay method [71].

The extracted proteins were used for the production of 2D gels. Briefly, for first dimension separation, 3 μg protein extract was passive-loaded into a 7 cm pH3-10 NL IPG strip (GE Healthcare), prior to being isoelectric focused for 9 kvh using the Ettan IPGphor III system (GE Healthcare). For the second dimension separation, the proteins from the strip were electrophoresed through a 12 % SDS polyacrylamide gel. The 2D gel was silver-stained [72] and it image was digitalized by Image Master Image Scanner III (GE Healthcare). Subsequently, spot analysis was performed on the digitalized image utilizing Image Master Platinum 7 software (GE Healthcare).

### Two dimensional differential gel electrophoresis (DIGE)

Mesocarp samples of 3 HY and 3 LY oil palms (biological replicates) at 12, 16 and 18 WAP were selected for initial DIGE screening.

### Setup of 2D-DIGE analytical gels

A total of 9 DIGE gels were produced for comparative analysis with sample pairing of HY and LY palm samples at each time point. An internal standard containing a pool of all 18 samples was included on the DIGE gels to aid peptide spot comparison between gels.

### CyDye labeling

For each sample, 30 μg of protein was mixed with 1.0 μL of diluted CyDye, and kept on ice in the dark for 30 min. Samples from each pair of HY and LY were labeled with Cy3 and Cy5, respectively, while the internal standard was labeled with Cy2. The labeling reaction was quenched with 1.0 μL of 10 mM lysine followed by further incubation for 15 min. The HY, LY and internal standard samples were then mixed together. Two times 2-D sample buffer (8 M urea, 4 % CHAPS, 20 mg/mL DTT, 2 % Pharmalyte and trace amount of bromophenol blue), Destreak solution (100 μL) and rehydration buffer (7 M urea, 2 M thiourea, 4 % CHAPS, 20 mg/mL DTT, 1 % Pharmalyte and trace amount of bromophenol blue) were added to the labeling mix to make a total volume of 250 μL. The resulting solution was mixed thoroughly and centrifuged prior to loading onto immobilized pH 4–7 gradient gel (IPG) strips.

### IEF (isoelectric focusing) and SDS-PAGE (polyacrylamide gel electrophoresis)

IEF was run following the protocol provided by Amersham BioSciences [73]. Once IEF was completed, the IPG strips were incubated in freshly prepared equilibration buffer-1 (50 mM Tris–HCl, pH 8.8, containing 6 M urea, 30 % glycerol, 2 % SDS, trace amount of bromophenol blue and 10 mg/mL DTT) for 15 min with gentle shaking. The strips were then rinsed in freshly prepared equilibration buffer-2 (50 mM Tris–HCl, pH 8.8, containing 6 M urea, 30 % glycerol, 2 % SDS, trace amount of bromophenol blue and 45 mg/mL DTT) for 10 min with gentle shaking. Lastly, the IPG strips were rinsed in SDS-gel running buffer before transferring into 12 % SDS-gels and run at 15 °C.

### Image scan and data analysis

Gel images were scanned immediately following SDS-PAGE using Typhoon TRIO (Amersham BioSciences). The scanned images were then analyzed using Image-Quant software (Version 6.0, Amersham BioSciences), followed by Biological Variation Analysis (BVA) using DeCyder™ 2D software Version 6.5 (Amersham BioSciences).

### Protein Identification by MALDI TOF/TOF mass spectrometer

Three preparative gels were prepared from running non-labeled proteins (ca 200 μg each) and spots were picked up from the gels by the aid of automated Ettan Spot Picker (Amersham BioSciences). The gel spots were washed, dried, and rehydrated in trypsin digestion buffer containing modified porcine trypsin protease (Trypsin Gold, Promega) at 37 °C. The trypsinized peptides were desalted by C18 Zip-tip (Millipore), eluted in matrix solution (5 mg/ml α-cyano-4-hydroxycinnamic acid in 50 % acetonitrile, 0.1 % trifluoroacetic acid, 25 mM ammonium bicarbonate) and spotted into wells of a MALDI plate. MALDI-TOF and TOF/TOF tandem MS were performed on an ABI 4700 mass spectrometer (Applied Biosystems, Framingham, MA). The mass spectra of MALDI-TOF were acquired in reflectron positive ion mode (ca. 4000 laser shots per spectrum). Tandem MS/MS fragmentation spectra of TOF/TOF were acquired (ca. 4000 laser shots per fragmentation spectrum) from 10 most abundant ions present in each sample. Protein identities were determined by searching against the database of National Center for Biotechnology Information non-redundant (NCBInr) with the combined MS and MS/MS spectra using GPS Explorer software equipped with the MASCOT search engine (Matrix Science). Searches were performed with carbamido methylation of cysteine and oxidation of methionine residues, and with allowance of one missed cleavage of trypsin. Hits with either protein score C.I.% greater than 80 or Ion C.I.% greater than 95 were considered significant. Precursor tolerance was set to 150 ppm and MS/MS tolerance was set to 0.4 Da.

### Validation of DIGE results using western analysis

Dot blot immunoassays were conducted on all 16 of the HY and LY samples collected at each of 12, 16 and 18 WAP (Additional file 4, Additional file 5, Additional file 6, Additional file 7). The specificity of antibodies used in the immunoassays was tested by 1D or 2D SDS-PAGE blots (Additional file 4; 1D and 2D SDS-PAGE methods are described in Additional file 5). Mesocarp proteins for dot blot immunoassays were extracted using the TCA-acetone method described by Carpentier et al. [74]. The resulting protein pellet was re-dissolved and stored in urea buffer (9 M urea, 4 % CHAPS, 1 % DTT, 1 % ampholytes pH3-10, 35 mM Tris base). The concentration of the protein extract was determined using the Bradford method [71]. Using a 386-pin replicator, protein samples were spotted onto nitrocellulose membranes. The membranes were allowed to dry overnight before incubation with a primary antibody. An alkaline phosphatase labeled secondary antibody was used for colorimetric detection with NBT/BCIP. Images of the membranes were scanned using Adobe Photoshop C84 Extended & Olympus Micro software to automatically capture and transform spot intensities into Microsoft Excel® data format. Comparisons between two populations were performed by using the Student *t*-test (Microsoft Excel® 2007). The receiver operating characteristic (ROC) curve was plotted using mROC (Version 1.0) by CIS Bio International.

## Additional files

Additional file 1:
**Two dimensional DIGE gels produced for spot analysis using 13 cm IEF strips of pH 4–7 with 20-kVh focusing.** Gel images of DIGE experiments labeled with Cy3 and Cy5 and their overlays. (PDF 2904 kb) Additional file 2:
**Data of Biological Variation Analysis (BVA) on DIGE gels.** Results of Biological Variation Analysis (BVA) on DIGE gels are given in a table. (PDF 95 kb) Additional file 3:
**Protein-peptide summary of MASCOT protein identification.** Protein-peptide summary of MASCOT protein identification. (XLSX 396 kb) Additional file 4:
**Validation of DIGE results by western analysis.** Results of western immunoblots carried out in this study. (PDF 193 kb) Additional file 5:
**Protocols for electrophoresis and immuno-blotting for western analysis, and antibodies used in this study.** Methods for 1D and 2D-SDS PAGE for immunoblots analysis and the list of antibodies used in this study. (PDF 77 kb) Additional file 6:
**Analysis of dot intensity of immunoblots for comparison of protein levels in mesocarp of high yielding and low yielding oil palms at 12, 16 and 18 week after pollination.** Analysis of dot intensity of immunoblots for comparison of protein levels in mesocarp of high yielding and low yielding oil palms at 12, 16 and 18 week after pollination. (PDF 120 kb) Additional File 7:
**Dot intensity analysis of immunoblots for time points comparison (12 vs 16 week after pollination and 12 vs 18 week after pollination) of mesocarp protein levels in high yielding oil palms.** Dot intensity analysis of immunoblots for time points comparison (12 vs 16 week after pollination and 12 vs 18 week after pollination) of mesocarp protein levels in high yielding oil palms (PDF 74 kb)

## Abbreviations

HY: High-yielding
LY: Low-yielding
WAP: Weeks after pollination
