# Supplementary material for: Differential abundance analysis of mesocarp protein from high- and low-yielding oil palms associates non-oil biosynthetic enzymes to lipid biosynthesis
Source: Proteome Sci. 2015 Nov 26;13:28. doi: 10.1186/s12953-015-0085-2 (PMC4661986; doi:10.1186/s12953-015-0085-2)
Supplement: Additional file 1: — Two dimensional DIGE gels produced for spot analysis using 13 cm IEF strips of pH 4–7 with 20-kVh focusing. Gel images of DIGE experiments labeled with Cy3 and Cy5 and their overlays. (PDF 2904 kb) [file 12953_2015_85_MOESM1_ESM.pdf]

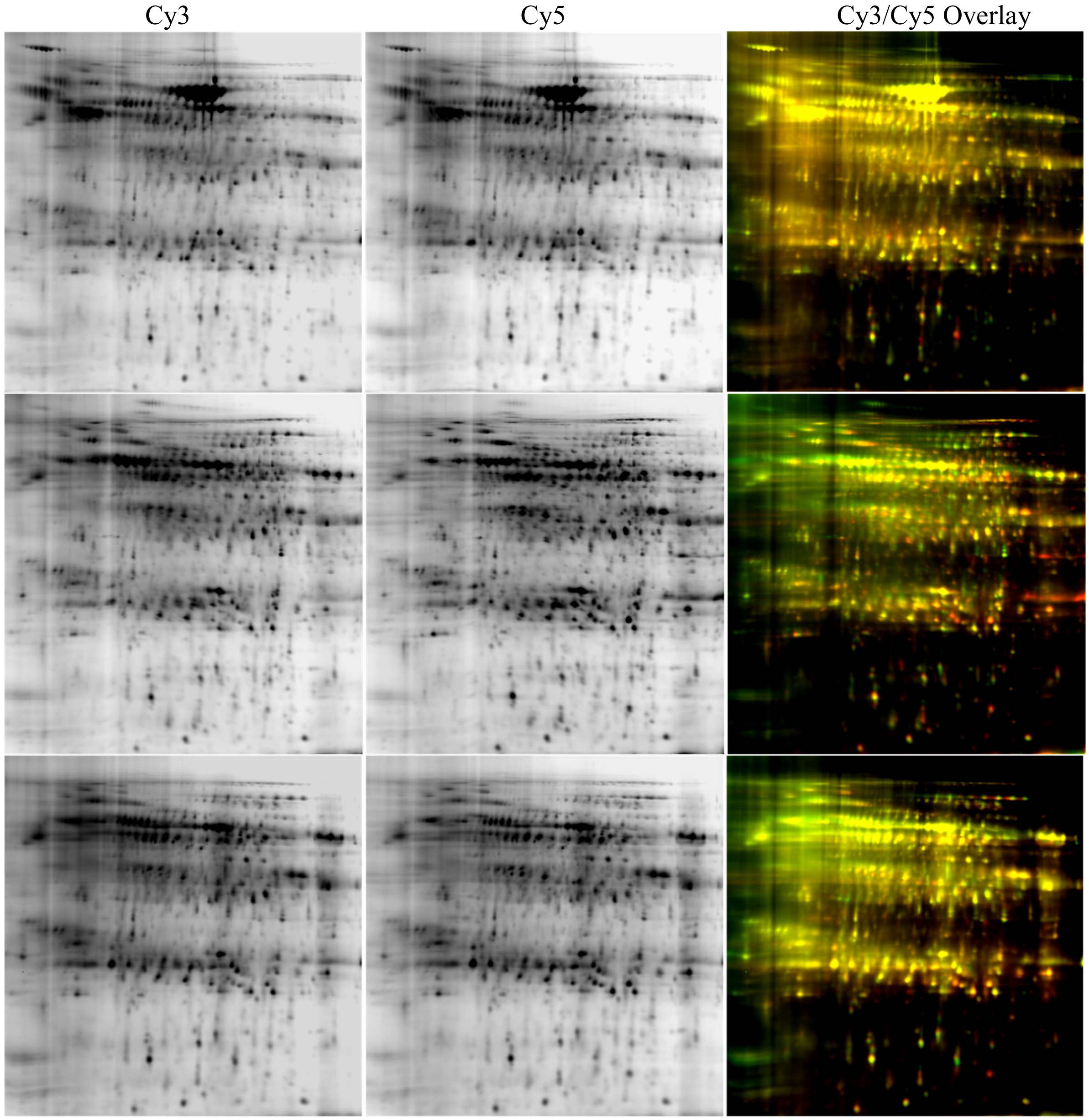

(A)

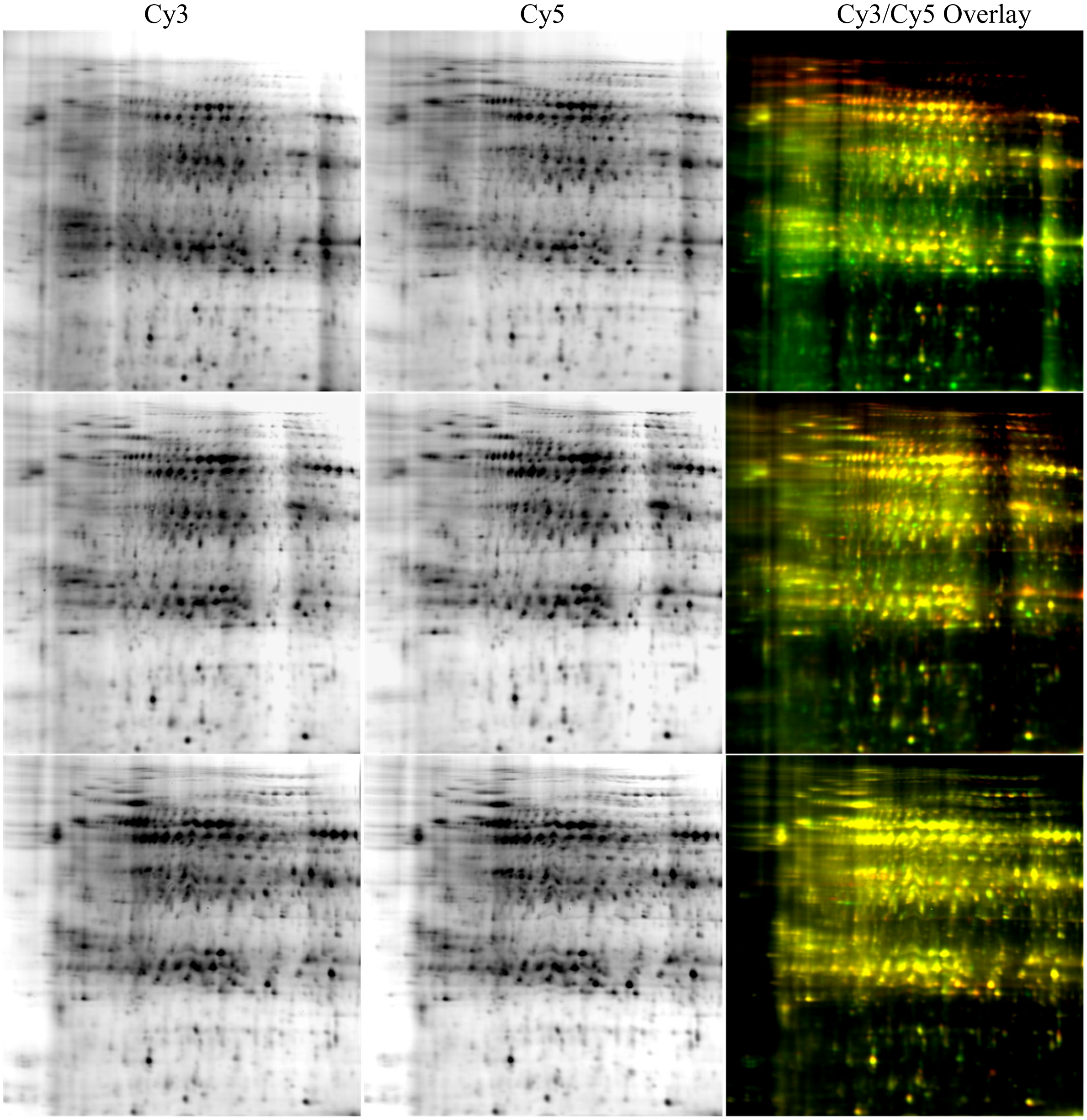

(B)

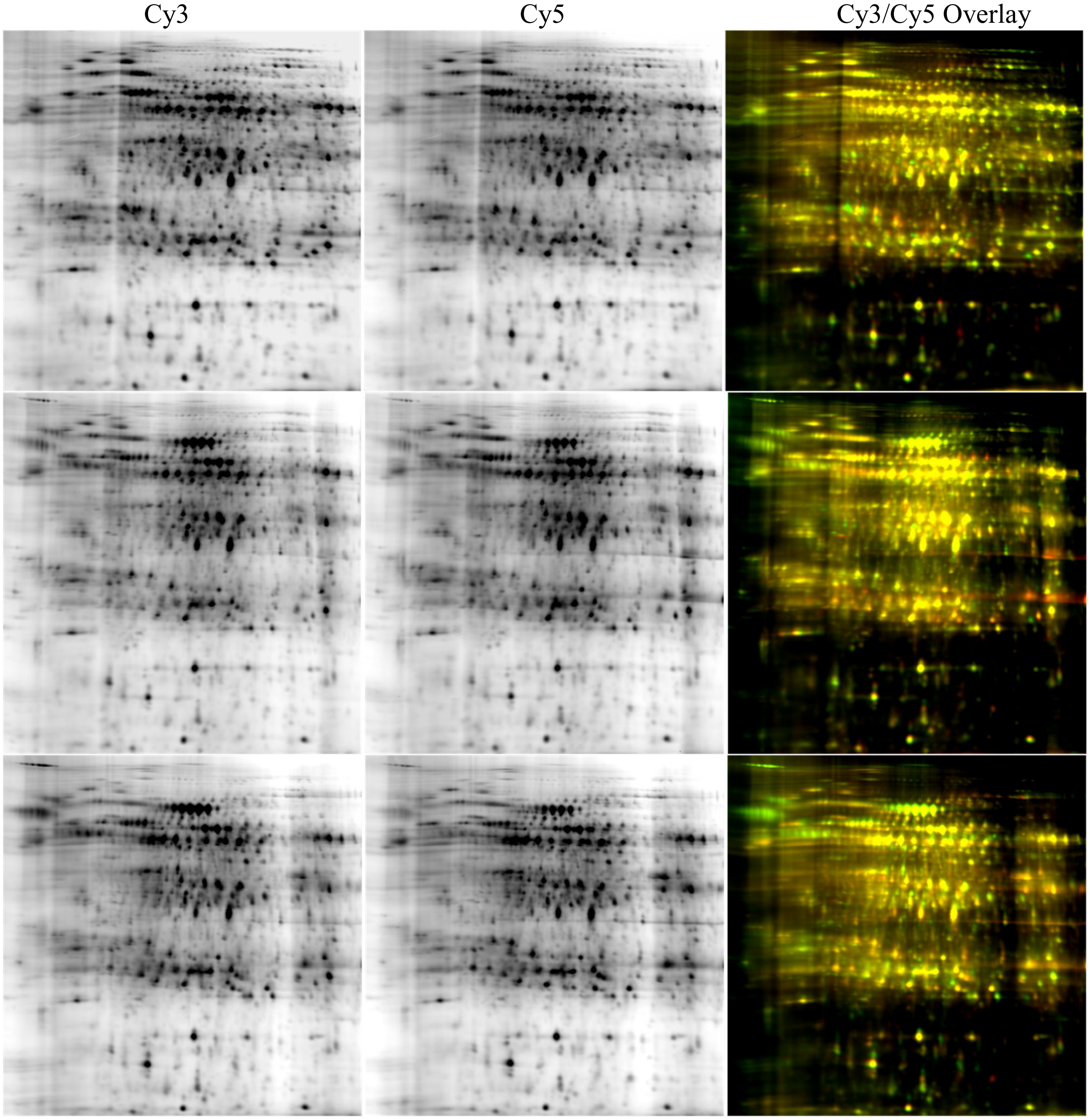

(C)

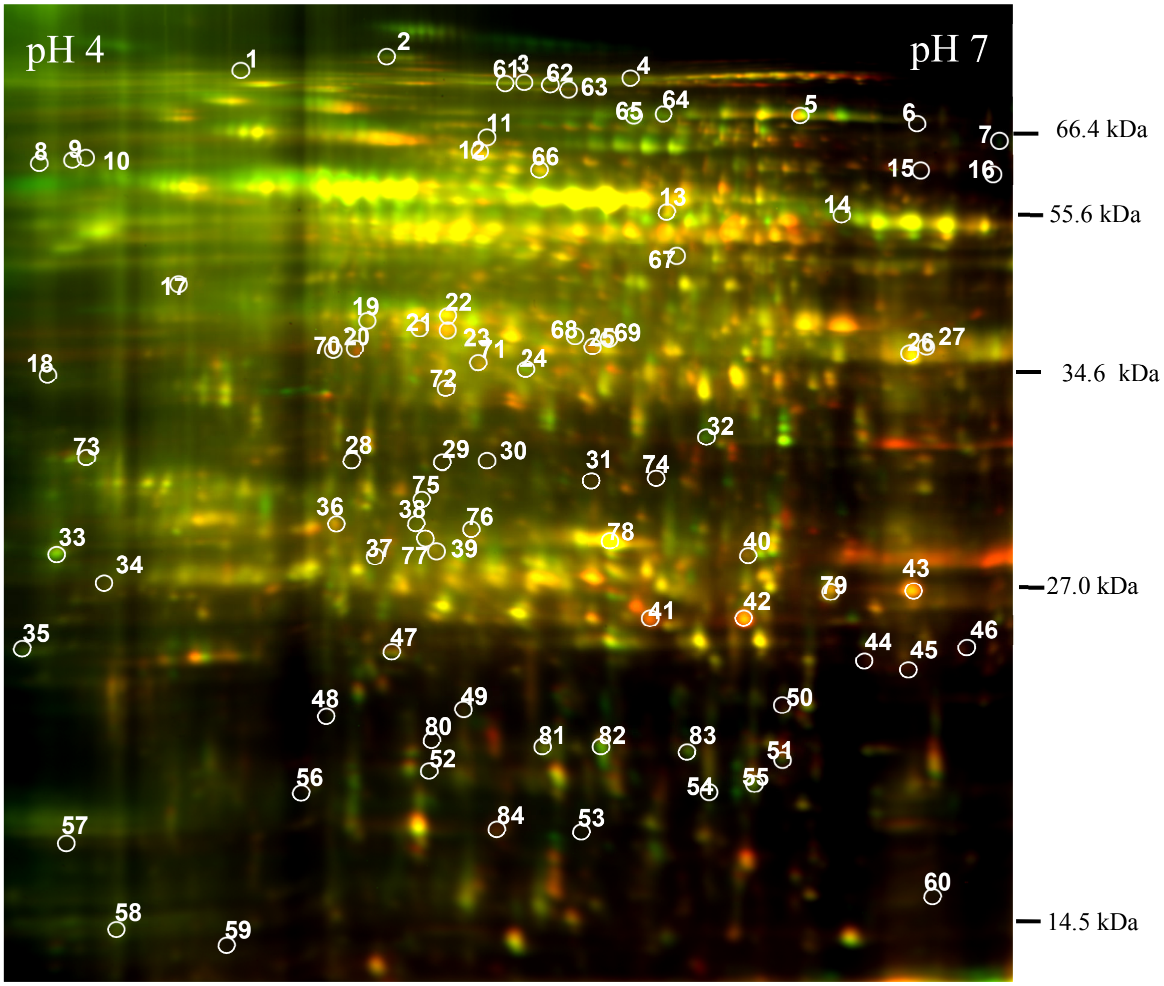

(D)

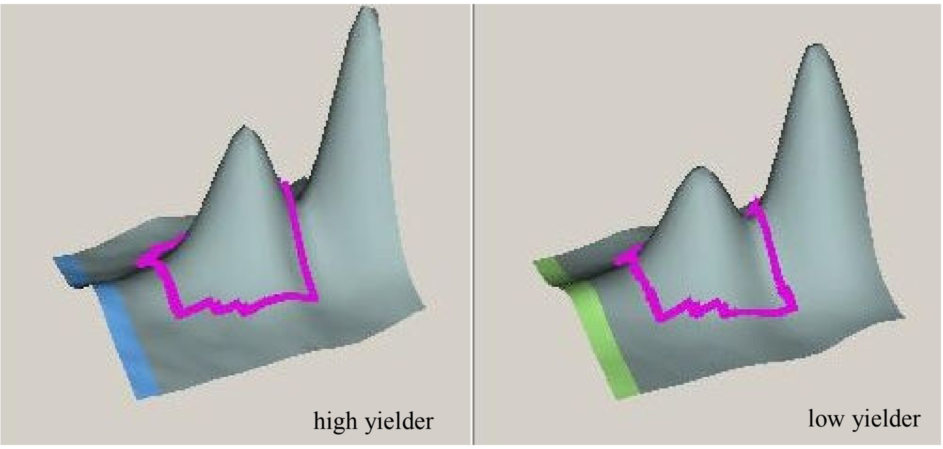

(E)

Additional File 1. Two dimensional DIGE gels produced for spot analysis using 13 cm IEF strips of pH 4 – 7 with 20-kVh focusing. Protein from high yielding palms was labeled with Cy3 whilst protein from low yielding palms with Cy5. Gels (A), (B) and (C) are from 3 biological replicates of 12, 16 and 18 WAP, respectively. Gel (D) is a composite of all DIGE gels. Numbers in Gel

(D) indicate the geographical position of protein spots in the map. Three-dimensional view (E) of a protein generated from a DIGE gel to verify that a spot is a true protein spot from an artifact. Illustrated here is the 3D view of protein Spot 80 (heat shock protein).
