# Supplementary material for: Differential abundance analysis of mesocarp protein from high- and low-yielding oil palms associates non-oil biosynthetic enzymes to lipid biosynthesis
Source: Proteome Sci. 2015 Nov 26;13:28. doi: 10.1186/s12953-015-0085-2 (PMC4661986; doi:10.1186/s12953-015-0085-2)
Supplement: Additional file 2: — Data of Biological Variation Analysis (BVA) on DIGE gels. Results of Biological Variation Analysis (BVA) on DIGE gels are given in a table. (PDF 95 kb) [file 12953_2015_85_MOESM2_ESM.pdf]

Additional File 2. Data of Biological Variation Analysis (BVA) on DIGE gels. Protein ratios are shown for 45 proteins found between low yielding (LY) palms and high yielding (HY) palms at 12, 16 and 18 week after pollination (WAP), and across time point among HY palms. Shown here are protein spots with differential ratio above 1.5 folds and with $p < 0.1$.

Protein Spot ID	Master No.	Appearance	LY/HY at 12 WAP		LY/HY at 16 WAP		LY/HY at 18 WAP		16/12 WAP of HY		18/12 WAP of HY	
			P value	Av.Ratio	P value	Av.Ratio	P value	Av.Ratio	P value	Av.Ratio	P value	Av.Ratio
63	277	27 (27)	0.9900	-1.02	0.0430	1.56	0.5100	-1.13	0.0270	-2.27	0.3300	-1.35
67	686	27 (27)	0.3400	-1.28	0.9000	-1.01	0.3000	-1.14	0.0360	1.79	0.0033	3.03
26	940	27 (27)	0.4500	1.17	0.0550	1.51	0.1600	1.20	0.0960	-1.36	0.8300	1.03
37	1393	27 (27)	0.5600	1.13	0.0370	1.61	0.7100	-1.07	0.4500	-1.18	0.3100	1.25
74	1200	27 (27)	0.5400	1.14	0.1600	-1.87	0.5900	-1.03	0.0310	3.46	0.0120	2.14
13	573	27 (27)	0.9600	1.03	0.0360	1.88	0.6000	-1.08	0.0080	-2.28	0.0031	-1.28
44	1631	27 (27)	0.0010	1.65	0.4400	1.10	0.1000	1.45	0.0110	1.51	0.8700	-1.01
46	1613	27 (27)	0.0043	1.50	0.9400	1.01	0.1800	1.45	0.0120	1.72	0.4000	-1.18
51	1831	27 (27)	0.1100	-1.47	0.0280	-1.88	0.3300	-1.41	0.0220	1.87	0.0100	2.80
75	1254	27 (27)	0.6800	-1.06	0.8700	-1.03	0.4900	1.13	0.0420	2.57	0.0013	5.50
5	345	27 (27)	1.0000	1.05	0.0690	1.87	0.7000	-1.07	0.2200	-1.70	0.8800	-1.09
21	862	27 (27)	0.2100	1.47	0.0630	1.77	0.3300	1.43	0.0200	-1.80	0.0100	-2.51
24	953	27 (27)	0.7700	-1.06	0.1800	-1.84	0.0130	-3.36	0.0210	4.22	0.0040	6.63
30	1184	27 (27)	0.7900	1.01	0.1200	-1.26	0.0720	-2.01	0.3900	1.24	0.5800	1.18
65	336	27 (27)	0.1400	-1.48	0.2200	1.35	0.1500	1.44	0.0085	-3.12	0.0039	-3.54
7	358	27 (27)	0.7400	-1.22	0.0250	1.72	0.3900	1.25	0.1300	-2.05	0.4600	1.17
41	1533	27 (27)	0.1600	2.39	0.0500	1.74	0.2300	1.45	0.0350	2.19	0.1500	1.79
43	1479	27 (27)	0.3500	2.10	0.0350	1.24	0.0980	-1.52	0.0480	2.26	0.0310	2.69

53	1895	24 (27)	0.2200	-1.12	0.0013	-1.87	0.3000	-1.16	0.0013	1.54	0.0330	1.26
54	1901	24 (27)	0.3600	-1.16	0.0300	-2.08	0.8300	1.16	0.2000	1.32	0.2000	-1.40
78	1367	27 (27)	0.0440	1.17	0.1200	1.44	0.5100	1.25	0.0120	-2.18	0.0140	-3.87
80	1799	27 (27)	0.7200	-1.12	0.1100	-1.56	0.6600	-1.07	0.0028	4.00	0.0008	6.37
22	826	27 (27)	0.6700	1.15	0.0860	1.64	0.5000	-1.16	0.1200	-1.60	0.3200	1.25
23	867	27 (27)	0.0370	2.09	0.0340	3.33	0.5000	1.43	0.0470	-2.05	0.0210	-2.08
68	892	27 (27)	0.6200	-1.09	0.1600	-1.22	0.8800	-1.01	0.0059	2.17	0.0006	3.61
73	1144	27 (27)	0.0044	-1.47	0.4000	1.19	0.5000	1.23	0.0220	-2.11	0.1700	-1.17
9	416	21 (27)	0.4800	-1.70	0.9700	1.01	0.0790	-2.16	0.2300	-2.98	0.6900	-1.54
12	460	21 (27)	0.5900	-1.17	0.0830	3.16	0.7100	-1.06	0.0430	-4.26	0.1900	-1.41
19	840	24 (27)	0.6600	1.15	0.0770	1.61	0.9100	-1.02	0.8200	1.08	0.1300	1.45
20	902	27 (27)	0.0990	1.56	0.0680	1.90	0.9700	-1.12	0.2100	-1.35	0.3600	1.62
32	1113	27 (27)	0.0610	-1.77	0.1700	-1.34	0.6400	1.16	0.0940	1.54	0.0610	1.52
33	1373	27 (27)	0.0110	-1.57	0.5800	1.08	0.0180	-1.54	0.0080	-2.01	0.0003	-1.62
34	1452	27 (27)	0.9400	1.05	0.0270	-1.63	0.9400	1.01	0.0280	1.58	0.0058	-1.34
36	1317	27 (27)	0.0090	1.65	0.6300	1.10	0.1000	1.11	0.0330	1.50	0.0120	1.41
39	1380	27 (27)	0.0610	1.68	0.3800	1.17	0.2100	1.25	0.0960	1.77	0.1300	1.58
48	1751	27 (27)	0.9000	1.02	0.0320	-1.85	0.5800	-1.14	0.0210	1.59	0.9400	1.01
49	1736	27 (27)	0.9200	1.00	0.4100	-1.74	0.0780	1.83	0.2500	2.08	0.0013	-2.47
55	1879	27 (27)	0.0900	-1.60	0.3200	-1.32	0.0014	-1.39	0.1200	-1.52	0.0200	-1.70
56	1886	27 (27)	0.4200	-1.11	0.1900	-1.22	0.0900	-1.64	0.0001	2.73	0.0003	7.01
58	2130	24 (27)	0.1500	-1.23	0.0690	-1.86	0.9100	1.10	0.4500	1.12	0.0220	-1.33
59	2200	27 (27)	0.7500	-1.03	0.0500	-1.83	0.7200	-1.10	0.0130	2.53	0.0027	3.28
66	501	27 (27)	0.8300	-1.09	0.1300	1.65	0.4200	1.06	0.0300	-2.56	0.2100	-1.47
72	995	27 (27)	0.2000	1.55	0.5300	-1.12	0.4200	1.13	0.0100	2.70	0.0160	2.24
79	1469	27 (27)	0.1600	1.49	0.5900	-1.10	0.5500	-1.07	0.0190	2.03	0.0650	1.49
84	1987	27 (27)	0.4800	1.44	0.4900	-1.22	0.7700	-1.04	0.0190	2.34	0.0018	2.62
