# Supplementary material for: Differential abundance analysis of mesocarp protein from high- and low-yielding oil palms associates non-oil biosynthetic enzymes to lipid biosynthesis
Source: Proteome Sci. 2015 Nov 26;13:28. doi: 10.1186/s12953-015-0085-2 (PMC4661986; doi:10.1186/s12953-015-0085-2)
Supplement: Additional file 4: — Validation of DIGE results by western analysis. Results of western immunoblots carried out in this study. (PDF 193 kb) [file 12953_2015_85_MOESM4_ESM.pdf]

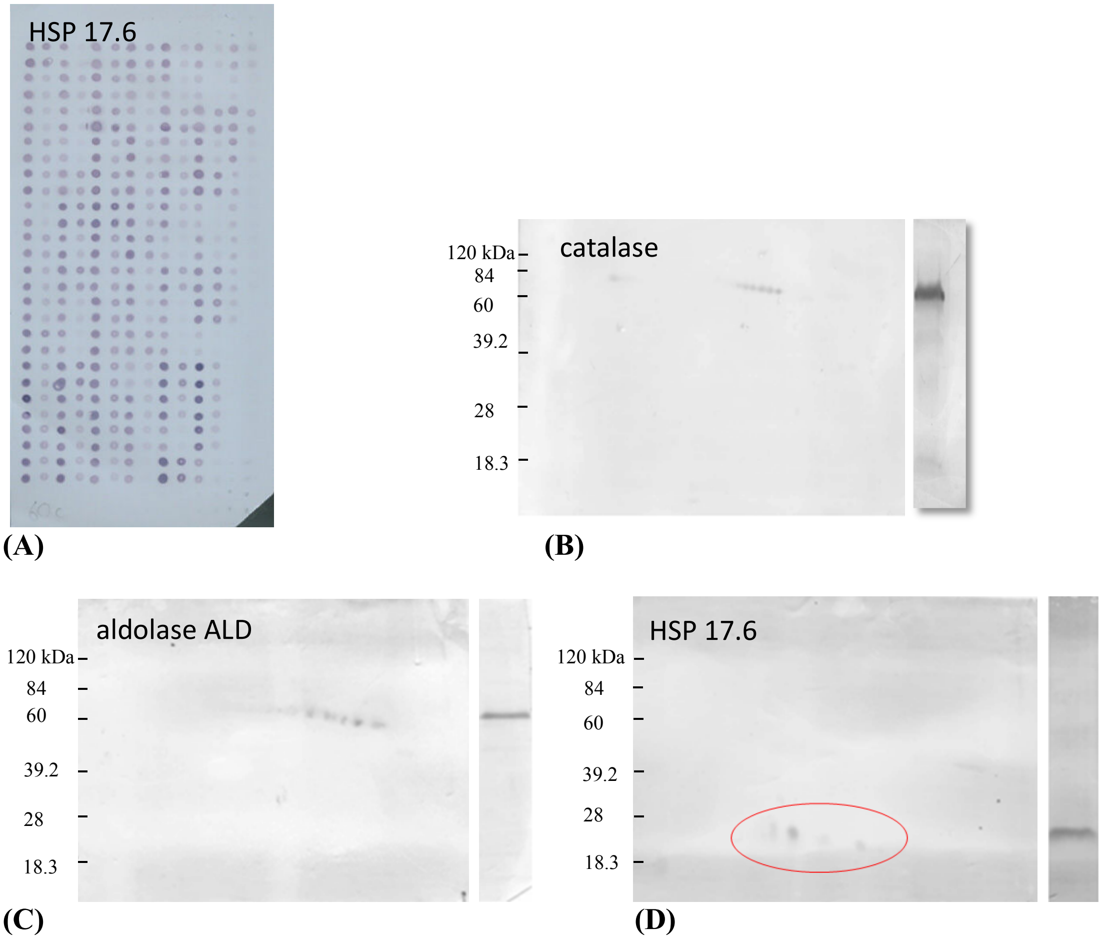

Additional File 4. Validation of DIGE results by western analysis. Dot blot immunoassays were conducted on all 16 of the high- and low-yielder samples collected at each of 12, 16 and 18 WAP. The immuno-dot blot of antibody HSP17.6C-CI is shown (A) as a representative. Prior to the dot blot immunoassay, specificity of antibodies used in the immunoassays was tested by 1DE or 2DE immuno-blots (See Additional File 5 for protocol). Results indicate that these antibodies were specific. (B), (C) and (D) are representative 2DE and 1DE immuno-blots produced in this study. They were probed with antibodies against catalase, fructose biphosphate aldolase and HSP17.6C-CI, respectively.
