# Supplementary material for: Differential abundance analysis of mesocarp protein from high- and low-yielding oil palms associates non-oil biosynthetic enzymes to lipid biosynthesis
Source: Proteome Sci. 2015 Nov 26;13:28. doi: 10.1186/s12953-015-0085-2 (PMC4661986; doi:10.1186/s12953-015-0085-2)
Supplement: Additional file 5: — Protocols for electrophoresis and immuno-blotting for western analysis, and antibodies used in this study. Methods for 1D and 2D-SDS PAGE for immunoblots analysis and the list of antibodies used in this study. (PDF 77 kb) [file 12953_2015_85_MOESM5_ESM.pdf]

Additional File 5. Protocols for electrophoresis and immuno-blotting for western analysis, and antibodies used in this study.

Protocols:

For 1DE, the Laemmli buffer system [Laemmli, U. K., Nature 1970, 222, 680–685] was used to cast 4.75% stacking and 12.5% resolving gel. After denaturation at 95°C for 3 min, proteins (30 µg) were electrophoresised under 200 V in a Bio-Rad mini-Protean III system until bromophenol blue dye reached the bottom of the minigel. For 2DE, protein samples (30µg) were applied in 125 µL of 2D rehydration solution by reswelling 7 cm IPG strips (pH 3–10 NL, GE Healthcare) overnight. IEF was performed in Ettan IPGphor II system following the protocol provided by Amersham BioSciences [*2-D electrophoresis: principles and methods*, GE Healthcare 2004]. Focused IPG strips were equilibrated using dithiothreitol and iodoacetamide solutions as described by Amersham BioSciences [*2-D electrophoresis: principles and methods*, GE Healthcare 2004] and then eletrophoresised onto a 12.5% minigel (1 mm thick) as above. After electrophoresis, proteins were visualized with Coomassie stain or electro-blotted onto nitrocellulose membranes. The protein blots were washed with 1X PBS, and blocked with 4% non-fat milk prior to incubating them with respective antibodies at the titer recommended by the suppliers. An alkaline phosphatase labeled secondary antibody was used for chromogenic detection with NBT/BCIP. The 1DE, 2DE and blot images were acquired using Image Scanner III (GE Heathcare).

List of Antibodies:

No.	**Spot No.**	**1° Antibody**	**Supplier of 1° Antibody**	**Host**	**1° Antibody Titer**	**2° Antibody**	**2° Antibody Titer**
1	9	AS09501: Catalase	Agrisera	Rabbit (Pab)	1:2000	DC067 Anti-Rabbit	1:5000
2	22	A0480-200ul: Anti-actin (plant), Clone 10-B3	Sigma	Mouse (Mab)	1:500	SAB-101 Anti-Mouse	1:5000
3	37	AB58329: Triosephosphate isomerase antibody	Abcam	Mouse (Mab)	1:500	AB5931 Anti-Mouse	1:5000
4	56	AS03037: RbcL, Rubisco large subunit, form I and form II	Agrisera	Rabbit (Pab)	1:5000	DC067 Anti-Rabbit	1:5000
5	74	AS08294: ALD, fructose-1,6 biphosphate aldolase	Agrisera	Rabbit (Pab)	1:5000	DC067 Anti-Rabbit	1:5000
6	5	AB9209: MTR antibody	Abcam	goat	1:1000	AB6742 Anti-Goat	1:5000
7	7	AB19104: HSP 90 antibody	Abcam	chicken	1:1000	AB6754 Anti Chicken	1:1000
8	13	AS09503: V-ATPase B, vacuolar H+ ATPase aubunit B	Agrisera	Rabbit (Pab)	1:1000	DC067 Anti-Rabbit	1:5000
9	21	AS07266: SMT1, sterol methyltransferase 1	Agrisera	Rabbit (Pab)	1:250	DC067 Anti-Rabbit	1:5000
10	23	AB51984: COMT	Abcam	Goat (Pab)	1:2000	AB6742 Anti-Goat	1:5000

11	24	GW22303B: Chicken Anti-XPNPEP (X-prolyl aminopeptidase (aminopeptidase P) 1) (N-193)	Sigma	chicken	1:500	AB6754 Anti Chicken	1:500
12	26	10494-1-AP: GAPDH	Proteintech	rabbit	1:500	DC067 Anti-Rabbit	1:5000
13	43	AB55188: glutathione s-transferase theta 1	Abcam	Mouse	1:500	SAB-101 Anti-Mouse	1:5000
14	49	AB18180: ABCA1 antibody [AB.H10]	Abcam	Mouse (Mab)	1:100	SAB-101 Anti-Mouse	1:5000
15	53	AB61866: Prostaglandin D Synthase (Lipocalin) antibody	Abcam	Rabbit (Pab)	1:500	DC067 Anti-Rabbit	1:5000
16	54	AS04055: GPX, chloroplastic glutathione peroxidase	Agrisera	Rabbit (Pab)	1:1000	DC067 Anti-Rabbit	1:10000
17	65	M4697-50: Anti-MTR (5-Methyltetrahydrofolate-homocysteine Methyltransferase)	USBiological	Goat	1:250	AB6742 Anti-Goat	1:5000
18	67	GTX110062: MCCC1	GeneTex	rabbit	1:500	DC067 Anti-Rabbit	1:5000
19	80	AS07254: HSP17.6 cytosolic class I HSP 17.6	Agrisera	Rabbit (Pab)	1:5000	DC067 Anti-Rabbit	1:5000
20	30	AB90146: HECT E3 ubiquitin ligase antibody	Abcam	Rabbit (Pab)	1:250	DC067 Anti-Rabbit	1:5000
21	32	AS07217: FtsZ, prokaryotic cell division GTPase	Agrisera	Rabbit (Pab)	1:1000	DC067 Anti-Rabbit	1:5000
22	33	ABIN337196: Nascent-polypeptide-associated+Complex+Alpha+Polypeptide+(NACA) (Human)	antibodies-online.com	rabbit	1:25	DC067 Anti-Rabbit	1:5000
23	41	AS09479: GST class-phi, glutathione S transferase	Agrisera	Rabbit (Pab)	1:500	DC067 Anti-Rabbit	1:5000
24	48	SC-102147: U2 snRNP A (F-22)	Santa Cruz Biotech	rabbit	1:100	DC067 Anti-Rabbit	1:5000
25	51	AS05093: PrxQ, peroxiredoxin, thioredoxin reductase	Agrisera	Rabbit (Pab)	1:250	DC067 Anti-Rabbit	1:5000
26	63	AS09556: PLD, phospholipase D	Agrisera	Rabbit (Pab)	1:1000	DC067 Anti-Rabbit	1:5000
27	68	ABIN229498 0: actin, Gamma 2, Smooth muscle enteric ACTG2	antibodies-online.com	rabbit	1:250	DC067 Anti-Rabbit	1:5000
28	72	17013-1-AP: RPL10	Proteintech	rabbit	1:1000	DC067 Anti-Rabbit	1:5000
