# Supplementary material for: Differential abundance analysis of mesocarp protein from high- and low-yielding oil palms associates non-oil biosynthetic enzymes to lipid biosynthesis
Source: Proteome Sci. 2015 Nov 26;13:28. doi: 10.1186/s12953-015-0085-2 (PMC4661986; doi:10.1186/s12953-015-0085-2)
Supplement: Additional file 6: — Analysis of dot intensity of immunoblots for comparison of protein levels in mesocarp of high yielding and low yielding oil palms at 12, 16 and 18 week after pollination. Analysis of dot intensity of immunoblots for comparison of protein levels in mesocarp of high yielding and low yielding oil palms at 12, 16 and 18 week after pollination. (PDF 120 kb) [file 12953_2015_85_MOESM6_ESM.pdf]

Additional File 6. Analysis of dot intensity of immunoblots for comparison of protein levels in mesocarp of high yielding and low yielding oil palms at 12, 16 and 18 week after pollination.

original	H or L	week	12_XPNPEP (chicken)	13_MTR (Goat)	15_Anti MTR (Goat)	41_COMT	47_L_ACTIN	48_TIM	51_ALD	53_L_ABCA1
Spot#			24	5	65	23	22	37	74	49
H2W12	H	12	186.3834519	149.2573789	198.3986331	75.58406068	101.0944811	87.0752424	58.68839938	247.1525766
H3W12	H	12	34.7812528	45.43009408	53.618348	6.67085328	37.59761264	28.6041392	20.59257872	99.13297184
H4W12	H	12	102.9805977	110.1083817	94.36788349	0	82.40666919	29.59890946	65.13398483	162.339294
H5W12	H	12	91.19115435	63.49150869	102.1191296	26.61763322	59.04085282	59.47098275	44.63786932	228.8714776
H6W12	H	12	88.44816469	74.24800406	92.83224844	14.986554	33.46956638	27.90338813	26.78588175	131.4080993
H7W12	H	12	97.58579388	72.09859505	169.900713	47.01018248	63.9742697	92.2491943	68.78978858	143.2811011
H8W12	H	12	193.8452448	158.5224219	358.7940711	187.8205536	187.6942002	166.8028485	132.6655326	374.2122063
H9W12	H	12	39.40141382	60.80953692	48.38186685	16.35150001	62.70891599	30.40480569	25.7024038	126.382746
L1W12	L	12	202.6025373	178.5264254	182.3604291	60.59658585	99.79808865	124.7202762	83.7045783	328.0965933
L3W12	L	12	160.0700672	104.6650268	257.363208	55.0287784	110.859858	96.9731924	87.0184228	305.9105592
L4W12	L	12	222.5765826	112.5808617	186.7234968	61.57633671	98.99421968	148.4457434	79.80564051	442.8826795
L5W12	L	12	138.3639753	83.16586624	193.4601677	108.9458324	105.4876453	128.032158	97.48348185	273.1774424
L6W12	L	12	225.1174151	135.3696189	283.6740731	85.47425129	145.0707453	129.2619625	90.55311691	301.7495093
L7W12	L	12	54.44359986	157.0079262	137.2773052	70.42267918	127.7182036	101.0348401	82.153352	314.831865
L8W12	L	12	120.0613809	140.9649326	265.1938548	89.56228549	112.5652028	244.2727279	121.0364311	373.9190083
L9W12	L	12	72.4164861	99.59413999	110.4490601	50.10986485	58.65621003	50.89427967	55.11382721	144.1161012
Mean HW12			104.3271342	91.74574016	139.8016117	46.88016716	78.498321	65.2636888	55.37455487	189.0975591
Mean LW12			149.4565055	126.4843497	202.0626993	72.71457676	107.3937717	127.9543975	87.10860633	310.5854698
Median HW12			94.38847411	73.17329956	98.24350655	21.48456661	63.34159285	44.93789422	51.66313435	152.8101976
Median LW12			149.2170212	123.9752403	190.0918322	65.99950794	108.1737516	126.3762171	85.36150055	310.3712121
T-test			0.084631919	0.044110029	0.084469502	0.146447412	0.084377579	0.01569797	0.025816032	0.007677895

original Spot#	H or L	week	54_L_V-ATPase 13	55_SMT1 21	56_GAPDH 26	57_lipocalin 53	58_L_GPX 54	60_HSP17.6 80	63_ACTG2 68	68_PrxQ 51
H2W12	H	12	143.8858879	83.54515675	166.7209481	334.3940602	82.46401648	75.94197171	195.0746498	191.8985043
H3W12	H	12	90.18216088	64.83957424	40.4361188	147.9456479	80.5245364	17.61356072	83.20785928	64.57827152
H4W12	H	12	136.8947526	171.1918429	94.66482511	305.2922037	117.8586394	25.03323764	124.3853555	112.6381258
H5W12	H	12	134.8957309	80.50924058	82.6706647	175.3464679	155.3159689	9.512590906	145.0647688	109.0113991
H6W12	H	12	91.294392	137.3619381	104.2337002	164.7527733	86.4995805	23.98730466	176.8643648	126.052821
H7W12	H	12	163.9418669	140.4017245	196.9495448	184.0453814	117.874779	33.60921541	199.0720671	178.5395426
H8W12	H	12	276.067077	380.5472436	376.5408089	490.8516224	288.9563823	102.9660653	444.4388975	387.7576935
H9W12	H	12	72.99800718	39.41220871	78.8243802	156.4980626	31.6671375	11.03412454	101.6657666	70.90312897
L1W12	L	12	184.6554629	191.8700111	227.422097	300.9558731	299.9876642	65.95211198	244.1587537	172.3667874
L3W12	L	12	202.759547	186.3479036	270.8239182	346.8513006	147.0548378	26.5651866	266.281623	254.132718
L4W12	L	12	186.6850373	144.7863173	98.04962772	252.4729333	160.8280998	43.15430543	225.6722516	162.4515077
L5W12	L	12	130.3324063	186.0573269	191.2582013	242.0526683	150.1389816	56.42016366	258.8668781	221.0695447
L6W12	L	12	245.7176886	336.0327398	229.3577255	265.5273355	491.997859	39.44740138	374.9704795	255.4128979
L7W12	L	12	163.5235123	78.33549523	85.29964342	305.4949938	73.03913516	49.68642417	162.9094118	123.7884089
L8W12	L	12	258.3178819	175.1718922	252.5839232	620.6377533	142.8723328	12.92702709	385.4769055	382.2781022
L9W12	L	12	121.8276607	100.628877	74.38156932	236.1027661	45.99718979	32.31718946	145.2861956	120.3944042
Mean HW12			138.7699844	137.2261162	142.6301238	244.8907774	120.14513	37.46225886	183.7217162	155.1724358
Mean LW12			186.7273996	174.9038204	178.6470882	321.261953	188.9895125	40.80872622	257.9528123	211.4867964
Median HW12			135.8952417	110.4535474	99.44926263	179.6959247	102.17911	24.51027115	160.9645668	119.3454734
Median LW12			185.6702501	180.6146096	209.3401491	283.2416043	148.5969097	41.3008534	251.5128159	196.718166
T-test			0.057624478	0.218573689	0.229989837	0.119879855	0.128937771	0.403636008	0.082862125	0.13044785

original Spot#	H or L	week	75_HECT 30	76_FtsZ 32	77_NACA 33	78_GST phi 41	79_U2-SnRNP 48	80_PLD 63	81_RPL10 72	83_Catalase 9	84_RbcL 56	85_MCCC1 67
H2W12	H	12	128.303738	166.5475289	52.36894433	248.4398193	45.1754441	78.04094775	34.34090351	268.7701131	78.64589148	0.26177065
H3W12	H	12	60.70783432	47.7215424	38.00046096	134.8919628	17.50974736	68.86161632	23.54281048	20.65782488	46.09663072	2.82152864
H4W12	H	12	26.17093364	84.42756096	23.32973046	209.2459223	18.64947429	110.9706901	8.627157656	142.3299915	90.26728935	0
H5W12	H	12	107.1576049	75.92472641	48.44025187	178.7771044	22.51250374	103.4320237	14.20750368	176.2002525	105.8013475	28.67401945
H6W12	H	12	47.05119684	74.73518306	30.94074356	176.9151127	28.56561413	117.4627322	24.18792328	212.0619931	86.95486088	22.15662675
H7W12	H	12	57.74781709	142.0846025	39.32074403	132.9422528	36.33992175	142.7134254	15.15842506	261.0885062	83.49870458	9.210003425
H8W12	H	12	270.2307833	314.0717634	74.415105	281.7699698	94.0406574	276.2072739	14.70670515	513.1701131	269.8236585	17.2724091
H9W12	H	12	32.46912319	41.04134335	12.65957403	111.7323342	12.97605036	33.32687006	8.784469986	23.19192242	37.43056516	0
L1W12	L	12	94.11741038	111.7487276	72.1071867	269.6439179	69.64358205	141.1502126	39.81431708	370.7939738	207.2321528	31.4114514
L3W12	L	12	115.611737	170.0250044	82.1643916	308.254529	40.8635616	143.7191788	30.1397594	168.114113	99.1310424	40.1713736
L4W12	L	12	182.1499515	158.118365	99.74141495	220.0212387	15.36216772	150.8409214	56.62593763	307.4069608	119.9799503	12.45657215
L5W12	L	12	121.7321541	166.4052986	56.5603899	131.2489291	44.07262136	173.3587465	21.53763156	269.8552653	110.5401517	0
L6W12	L	12	132.7683521	241.7206706	82.19143034	289.0528383	65.45977321	241.7875341	32.29635858	424.4177235	200.8345203	13.5920306
L7W12	L	12	140.6284007	108.7697771	32.46973251	197.3242219	26.52019333	90.92701821	22.95818582	191.7389173	133.6883104	0
L8W12	L	12	102.2556372	152.4986949	122.1302993	436.2401632	12.04195676	110.8480351	49.48980107	48.91551407	93.85508081	0
L9W12	L	12	44.73306268	100.4463144	35.39232216	184.0355327	35.81452507	109.5368946	13.77381721	48.78580068	57.26131115	0.591601992
Mean HW12			91.22987891	118.3192814	39.93444428	184.3393098	34.47117664	116.3769474	17.94448735	202.1838396	99.81486852	10.04954475
Mean LW12			116.7495882	151.2166066	72.84464593	254.4776714	38.72229764	145.2710677	33.32947604	228.7535336	127.815315	12.27787872
Median HW12			59.2278257	80.17614368	38.66060249	177.8461085	25.53905893	107.2013569	14.93256511	194.1311228	85.22678273	6.015766033
Median LW12			118.6719456	155.3085299	77.13578915	244.8325783	38.33904334	142.4346957	31.21805899	230.7970913	115.260051	6.524087073
T-test			0.2198104	0.18948313	0.012399523	0.05011231	0.36351961	0.182264512	0.012941381	0.363223013	0.195505016	0.37519975

original Spot#	H or L	week	12_XPNPEP (chicken) 24	13_MTR (Goat) 5	15_Anti MTR (Goat) 65	41_COMT 23	47_L_ACTIN 22	48_TIM 37	51_ALD 74	53_L_ABCA1 49
H2W16	H	16	345.7682685	352.9520037	229.9578685	101.4742244	106.4645301	110.5816078	220.0007821	304.016059
H3W16	H	16	121.961944	89.65175803	140.5605812	34.57684425	47.68071963	55.01264579	82.75657021	194.6733597
H4W16	H	16	356.4755102	177.0378146	200.5694668	0	126.531695	49.64953397	197.7346008	359.3538746
H5W16	H	16	296.8275657	115.3765807	246.0181785	52.31238853	47.12765633	106.6407974	190.3857164	262.2429787
H6W16	H	16	478.4295847	240.0235721	385.9712519	28.40444228	150.2715206	95.78728912	218.6304148	558.6203492
H7W16	H	16	238.5105008	70.26186225	183.4327794	92.33571075	121.4975128	113.2950846	118.9975569	282.0054623
H8W16	H	16	50.74442078	98.94554797	72.7414275	71.75050281	76.53837672	72.26861438	69.23182422	194.9244756
H9W16	H	16	264.0959005	183.7812376	230.4884977	74.69194584	165.0267269	133.6396682	193.126268	320.0927834
L1W16	L	16	406.2749774	176.9842767	263.5962937	134.2884543	127.8455228	215.3070761	183.520873	516.7381841
L3W16	L	16	368.8700445	190.8734411	430.6235089	84.9998283	211.003393	171.8225408	137.8059521	617.0955692
L4W16	L	16	407.7981073	178.6270168	267.6137672	85.3027032	152.7867872	182.557028	139.4077784	489.2554141
L5W16	L	16	276.4129046	121.5367136	218.8132467	130.9345447	101.5163507	145.4190364	156.1780123	238.0859687
L6W16	L	16	226.9763158	148.510674	343.4926088	71.403903	105.398122	96.354455	102.9167388	367.8523913
L7W16	L	16	55.78181472	139.2402327	132.4181061	62.34166033	120.5174162	80.33109224	150.9752219	249.4394577
L8W16	L	16	85.6524093	84.1010136	168.6859841	54.3204796	67.3700649	124.0393345	85.1684524	243.9148084
L9W16	L	16	300.4793983	281.822153	369.6829194	25.604216	171.569353	132.5487547	221.5816826	383.9573958
Mean HW16			269.1017119	166.0037971	211.2175064	56.94325736	105.1423423	92.10940516	161.3579667	309.4911678
Mean LW16			266.0307465	165.2119402	274.3658043	81.14947368	132.2508762	143.5474147	147.1943389	388.2923987
Median HW16			280.4617331	146.2071976	215.2636676	62.03144567	113.9810214	101.2140433	191.7559922	293.0107606
Median LW16			288.4461515	162.7474754	265.6050304	78.20186565	124.1814695	138.9838955	145.1915002	375.9048935
T-test			0.482286052	0.492152732	0.105986721	0.098873615	0.122875021	0.009757667	0.301029744	0.12292352

original Spot#	H or L	week	54_L_V-ATPase 13	55_SMT1 21	56_GAPDH 26	57_lipocalin 53	58_L_GPX 54	60_HSP17.6 80	63_ACTG2 68	68_PrxQ 51
H2W16	H	16	132.6018009	179.0566214	101.5211664	421.0777045	122.1860864	88.75921829	197.870842	220.4594102
H3W16	H	16	138.6439864	207.4224283	251.1901827	309.6608579	146.9685781	26.69821896	204.2935913	170.2917341
H4W16	H	16	184.0753036	199.2266762	265.0093873	439.1006298	146.1013814	31.68221346	315.0697118	199.9105548
H5W16	H	16	143.2192588	188.9004686	142.0963089	329.6837561	150.1423625	23.29807586	273.918601	214.9392523
H6W16	H	16	228.1596087	382.2641558	339.5184806	522.8521218	178.6487535	69.85209309	582.3561248	283.6457015
H7W16	H	16	154.2650458	168.207513	175.5208	196.8973604	138.4859033	47.19144534	224.2214326	207.3118001
H8W16	H	16	94.79997172	81.13146734	46.57846023	200.9714324	29.294585	45.55035664	132.5013912	97.22181422
H9W16	H	16	156.0894618	176.880298	250.8472315	504.8360739	86.76493482	37.55759917	387.8327877	323.4108906
L1W16	L	16	177.4719887	319.1875368	452.829486	360.8810374	133.7668811	63.33496744	444.8352154	325.308786
L3W16	L	16	334.2698705	364.2798182	256.0278051	523.0038441	315.3860047	36.50059349	405.1433699	413.3734518
L4W16	L	16	217.373272	202.6417	147.4407716	350.5861804	212.8433952	57.4965924	319.092122	240.2882016
L5W16	L	16	132.2957719	196.1650175	246.1543767	281.4602634	144.2049645	66.46448392	365.5734226	288.0238319
L6W16	L	16	165.9484643	332.3668703	394.7284936	313.7161359	99.851649	32.39053963	447.7499301	281.4169108
L7W16	L	16	144.347248	86.44196946	35.29303852	282.969648	67.62262474	23.54551996	186.8457591	137.9110775
L8W16	L	16	174.7825539	209.7910034	103.890631	291.0170052	147.7552795	9.44299835	204.2873373	165.7948492
L9W16	L	16	182.3035615	189.5783027	229.1982123	788.9769684	75.59559721	65.11796644	289.8724549	406.3582497
Mean HW16			153.9818047	197.8862036	196.5352522	365.6349921	124.8240731	46.3236526	289.7580603	214.6488947
Mean LW16			191.0990913	237.5565273	233.1953519	399.0763853	149.6282995	44.2867077	332.9249514	282.3094198
Median HW16			148.7421523	183.978545	213.1840158	375.3807303	142.2936423	41.5539779	249.0700168	211.1255262
Median LW16			176.1272713	206.2163517	237.6762945	332.1511581	138.9859228	46.99859294	342.3327723	284.7203713
T-test			0.092073786	0.193042622	0.277382963	0.33521369	0.235164239	0.428373456	0.248220866	0.070227135

original Spot#	H or L	week	75_HECT 30	76_FtsZ 32	77_NACA 33	78_GST phi 41	79_U2-SnRNP 48	80_PLD 63	81_RPL10 72	83_Catalase 9	84_RbcL 56	85_MCCC1 67
H2W16	H	16	210.1532269	205.9253231	99.90496085	391.5711896	60.86890893	325.5571206	45.05767734	155.6390693	216.1014718	0
H3W16	H	16	52.50873099	135.1484409	66.96585705	283.2002405	32.71829144	129.3490662	29.02300548	122.0910509	81.11086698	25.70667456
H4W16	H	16	65.39122478	202.4841724	84.36570259	382.9227193	55.82940785	204.4117474	66.88973481	243.7409434	170.6528029	19.80159254
H5W16	H	16	93.75830826	212.2958222	86.3929247	175.7835244	45.5351558	136.8488907	26.76859591	330.3777869	110.8185261	25.63686082
H6W16	H	16	264.8805114	221.006347	133.1139481	511.1897865	95.24763751	269.7930155	66.72581142	710.4401851	301.356879	102.2163643
H7W16	H	16	60.95847009	149.6452813	39.44159531	185.0339718	32.42442506	128.1478489	24.55726191	314.1117578	87.51519581	0
H8W16	H	16	96.29887992	82.95622516	39.20810531	207.0035091	20.69403563	58.81446563	13.56145477	166.1127463	85.64413188	0
H9W16	H	16	41.60947231	145.0109238	70.30012399	386.0573589	22.57513403	129.0064001	35.59159131	72.93515938	72.92582859	0
L1W16	L	16	118.5436325	271.1686634	111.2351897	353.8496292	43.5598317	275.245127	45.83577529	443.6017356	307.0247763	9.17532
L3W16	L	16	253.671607	325.5225341	184.7934791	522.0812434	58.5632905	243.4181996	73.66299771	388.0429995	188.778148	64.11523033
L4W16	L	16	159.4307604	223.514412	122.8067208	260.095786	22.8569808	185.7372072	52.9858196	366.125374	163.2799776	7.9561792
L5W16	L	16	157.7501674	200.8136728	43.74416018	191.5513794	35.82315463	153.4394088	9.302171981	314.3579455	145.0967121	0
L6W16	L	16	186.5064609	249.1609465	80.13478	338.2340791	78.1606715	269.438315	56.64664738	469.8732641	248.612555	41.0849665
L7W16	L	16	130.4333004	96.61559284	57.95064521	225.3012577	14.60744711	112.9886342	10.32768218	108.953296	74.9718716	0
L8W16	L	16	70.85032145	85.4184592	64.5292233	220.4743487	26.0334841	83.3478084	14.31189405	47.66630445	32.0307279	0
L9W16	L	16	43.05134082	235.1102094	123.9955353	421.903329	82.7701644	238.2452138	46.51332819	176.6730281	176.003469	17.00594981
Mean HW16			110.6948531	169.309067	77.46165224	315.3452875	45.73662453	172.7410694	38.52189162	264.4310874	140.7657129	21.67018652
Mean LW16			140.0296989	210.9155613	98.6487167	316.6863816	45.29687809	195.2324893	38.69828955	289.4117434	166.9747797	17.41720573
Median HW16			79.57476652	176.0647268	77.33291329	333.0614799	39.12672362	133.0989784	32.30729839	204.9268449	99.16686094	9.900796269
Median LW16			144.0917339	229.3123107	95.68498486	299.1649326	39.69149316	211.9912105	46.17455174	340.2416597	169.6417233	8.5657496
T-test			0.22184	0.122463854	0.151686214	0.491084419	0.486304149	0.293019166	0.493742215	0.393641791	0.273831042	0.389069354

original Spot#	H or L	week	12_XPNPEP (chicken) 24	13_MTR (Goat) 5	15_Anti MTR (Goat) 65	41_COMT 23	47_L_ACTIN 22	48_TIM 37	51_ALD 74	53_L_ABCA1 49
H2W18	H	18	452.9165137	300.1827442	233.9870369	45.57052849	80.83512091	72.749376	299.1531655	196.735008
H3W18	H	18	661.9366492	222.5757718	461.3325974	83.76839775	147.4757523	157.9676132	479.4000229	566.0965656
H4W18	H	18	1289.905455	355.7164755	656.3448286	49.32793016	295.2194832	205.7054952	799.6133038	704.4455362
H5W18	H	18	1247.414948	516.9411764	898.4835551	277.8705386	228.1995934	331.7984207	1047.558666	809.1616081
H6W18	H	18	540.4257914	243.9880663	304.8942707	36.4140601	142.0490099	78.7722859	302.5707693	456.2348681
H7W18	H	18	696.5217883	211.5899305	584.6350686	316.09188	226.5305913	308.0960116	469.8149495	497.5412449
H8W18	H	18	63.0353352	283.3445073	296.9491749	119.364555	224.1796596	227.6028339	407.1345969	464.4339336
H9W18	H	18	287.995701	106.8340587	181.4721066	36.8064849	96.7965348	118.6674504	274.4139885	289.5481986
L1W18	L	18	1062.037083	384.013	757.347615	390.2741061	320.6141479	482.7438718	890.1240628	1102.37313
L3W18	L	18	195.0866671	79.339	165.0726067	63.19339683	130.1919154	110.4918084	172.6255629	339.9497754
L4W18	L	18	324.8721361	98.56097366	254.7149732	48.55154543	144.4119027	140.7720667	230.9645872	380.3536812
L5W18	L	18	488.3038173	195.4291103	464.8847241	239.4640944	307.0181986	237.3040004	413.2928089	354.4657886
L6W18	L	18	579.8609006	438.5459296	679.0814747	427.6768901	222.4801258	267.7024702	675.2024182	652.1785943
L7W18	L	18	221.5436108	168.7199889	263.6957589	76.23613549	205.1414869	127.0455597	279.7289235	326.7052257
L8W18	L	18	440.679449	165.650737	370.7714966	115.2695896	155.6642658	195.2420408	368.1687188	393.3753492
L9W18	L	18	346.161843	143.1549405	164.8560582	44.7301539	69.1929063	60.665418	166.8325626	195.5221389
Mean HW18			655.0190227	280.1465913	452.2623298	120.6517969	180.1607182	187.6699359	509.9574329	498.0246204
Mean LW18			457.3181883	209.17671	390.0530884	175.674489	194.3393687	202.7459045	399.6174556	468.1154605
Median HW18			601.1812203	263.6662868	383.1134341	66.54816396	185.827706	181.8365542	438.4747732	480.9875892
Median LW18			393.420646	167.185363	317.2336277	95.75286254	180.4028764	168.0070537	323.9488211	367.4097349
T-test			0.147903525	0.139325503	0.303804752	0.218084198	0.366103979	0.399931687	0.210892142	0.406286101

original<br>Spot#	H or L	week	54_L_V-ATPase<br>13	55_SMT1<br>21	56_GAPDH<br>26	57_lipocalin<br>53	58_L_GPX<br>54	60_HSP17.6<br>80	63_ACTG2<br>68	68_PrxQ<br>51
H2W18	H	18	41.04331425	163.0462461	134.2222153	415.6210688	256.1539901	55.50195953	166.9849202	206.3668125
H3W18	H	18	235.1024169	435.9271885	319.2086542	681.7140834	233.5909497	50.04840028	514.2502029	481.5719785
H4W18	H	18	250.1384383	789.7965928	324.7169406	954.2869278	227.7152907	51.69066781	867.8765562	636.9702195
H5W18	H	18	321.4416875	766.7318222	534.1618356	1178.670105	307.9543484	103.5684053	931.3168008	946.8603113
H6W18	H	18	71.75046478	348.6611596	238.1214133	447.5581433	0	50.90544924	338.8159672	218.3728605
H7W18	H	18	243.0770271	487.5871065	502.0533411	437.7159537	237.4886995	114.1233005	564.5026371	636.8514347
H8W18	H	18	207.458258	96.3212718	229.6302017	687.2214972	30.03434325	91.97596185	588.1372731	468.8349018
H9W18	H	18	149.2383224	174.964155	270.6918509	363.1911935	97.83772605	35.96868915	299.1316487	299.8248663
L1W18	L	18	429.4360907	861.5331655	1380.63008	757.1683148	402.8093069	157.3044311	1259.663839	767.2483737
L3W18	L	18	168.8630275	148.2365209	73.06567694	284.8505317	135.4613085	29.98051631	226.5467158	185.5893221
L4W18	L	18	178.5054356	231.5876691	149.2633936	271.7737115	145.528195	57.17871276	298.1753312	242.1999583
L5W18	L	18	201.1215436	474.9956055	279.3233589	509.9046208	252.8447442	91.60230747	689.6814626	527.2178539
L6W18	L	18	346.4194288	371.1721998	313.517715	933.0062677	229.5724533	121.422368	618.0965353	630.6940855
L7W18	L	18	155.0473228	145.0090451	59.57026928	477.2469781	61.75551632	8.924149456	333.7544889	246.4369049
L8W18	L	18	215.3650669	365.5147188	304.0561735	607.0094773	279.7150125	11.3340397	501.8242915	428.3261406
L9W18	L	18	103.6409522	139.6052703	83.71733265	435.4995272	70.72939395	42.37343145	254.2397414	249.3569475
Mean HW18			189.9062411	407.8794428	319.1008066	645.7473716	173.8469185	69.22285421	533.8770008	486.9566731
Mean LW18			224.7998585	342.2067744	330.3929999	534.5574286	197.3019913	65.01499453	522.7478007	409.6336983
Median HW18			221.2803374	392.294174	294.9502525	564.6361134	230.6531202	53.59631367	539.37642	475.2034402
Median LW18			189.8134896	298.551194	214.2933763	493.5757995	187.5503242	49.7760721	417.7893902	338.8415441
T-test			0.252856865	0.307746931	0.47304312	0.205012744	0.345093279	0.424754155	0.471734552	0.259522787

original	H or L	week	75_HECT	76_FtsZ	77_NACA	78_GST phi	79_U2-SnRNP	80_PLD	81_RPL10	83_Catalase	84_RbcL	85_MCCC1
Spot#			30	32	33	41	48	63	72	9	56	67
H2W18	H	18	157.974246	189.914303	97.12253701	438.392237	77.77209613	255.6255557	52.90331646	183.0285303	196.9639286	14.87780672
H3W18	H	18	205.6824297	343.1686751	156.6612194	545.864324	77.25891113	301.3540493	82.65325397	519.409243	225.3717583	58.847266
H4W18	H	18	244.4811829	647.4847629	279.2768194	924.2951056	159.3876024	516.3575027	139.2255923	736.5038514	415.0161906	110.7837068
H5W18	H	18	219.4298165	702.8104818	206.8075368	953.2922445	222.818115	408.3595783	100.7323562	1405.585531	286.9133979	26.28105594
H6W18	H	18	149.7310998	146.2072928	139.0358197	266.6501309	59.6017873	228.717654	53.68972826	578.3066827	217.0362024	35.271654
H7W18	H	18	320.6667844	411.4686953	168.088843	556.065509	159.1566197	336.6506702	22.87259942	747.2754604	271.5233736	0
H8W18	H	18	155.8890341	283.2516156	119.5186068	432.3349892	49.424526	234.1733223	22.07303835	254.9420384	157.823766	0
H9W18	H	18	61.87299915	116.7747276	90.7209087	255.5745464	31.0342791	141.7434183	25.73005905	82.98386715	64.7104104	0
L1W18	L	18	355.8589175	753.1443668	339.3805244	884.6815256	217.6783338	714.6227804	150.4164803	1158.218503	622.7668708	46.52825747
L3W18	L	18	187.8466917	156.9109571	99.6978541	298.4512081	27.47766255	142.6055521	25.87997344	317.3644356	111.5768859	23.3321998
L4W18	L	18	152.6041064	184.5573987	67.4262225	184.4014567	35.14478648	196.3298196	17.25755916	354.7604352	119.6698819	4.185941737
L5W18	L	18	368.7852788	418.1384104	112.0207266	445.3785078	122.4599574	306.3011573	29.77474897	591.1920516	308.1660628	0
L6W18	L	18	273.079162	529.9551453	211.8720759	956.5359226	142.7172447	327.1910836	27.16670684	533.3709234	197.2509832	13.42001669
L7W18	L	18	148.5314603	149.1644915	76.0487721	331.4376755	17.88768313	147.3892358	13.70447004	129.21474	89.90476425	0
L8W18	L	18	232.7697335	207.5181178	124.4683536	451.5384227	47.7462562	175.4907074	56.5735929	412.9147607	118.295415	65.2793764
L9W18	L	18	43.46094465	101.991888	59.7292173	245.7018428	56.0686653	119.289933	20.94104475	126.7631969	77.4310851	0
Mean HW18			189.4659491	355.1350693	157.1540364	546.5586358	104.5567421	302.8727188	62.484993	563.5044005	229.4198785	30.75768619
Mean LW18			220.3670368	312.672597	136.3304683	474.7658202	83.39757369	266.1525336	42.71432205	452.9748808	205.6327436	19.09322401
Median HW18			181.8283379	313.2101453	147.8485196	492.1282805	77.51550363	278.4898025	53.29652236	548.8579628	221.2039803	20.57943133
Median LW18			210.3082126	196.0377583	105.8592903	388.4080916	51.90746075	185.9102635	26.52334014	383.837598	118.9826484	8.802979212
T-test			0.263924118	0.356624941	0.306924934	0.307134531	0.274893967	0.329256689	0.191304577	0.28469511	0.377853273	0.241804834
