# Supplementary material for: Differential abundance analysis of mesocarp protein from high- and low-yielding oil palms associates non-oil biosynthetic enzymes to lipid biosynthesis
Source: Proteome Sci. 2015 Nov 26;13:28. doi: 10.1186/s12953-015-0085-2 (PMC4661986; doi:10.1186/s12953-015-0085-2)
Supplement: Additional File 7: — Dot intensity analysis of immunoblots for time points comparison (12 vs 16 week after pollination and 12 vs 18 week after pollination) of mesocarp protein levels in high yielding oil palms. Dot intensity analysis of immunoblots for time points comparison (12 vs 16 week after pollination and 12 vs 18 week after pollination) of mesocarp protein levels in high yielding oil palms (PDF 74 kb) [file 12953_2015_85_MOESM7_ESM.pdf]

Additional File 7. Dot intensity analysis of immunoblots for time points comparison (12 vs 16 week after pollination and 12 vs 18 week after pollination) of mesocarp protein levels in high yielding oil palms.

original Spot#	H or L	week	12_XPNPEP (chicken) 24	13_MTR (Goat) 5	15_Anti MTR (Goat) 65	41_COMT 23	47_ACTIN 22	48_TIM 37
H2W12	H	12	186.3834519	149.2573789	198.3986331	75.58406068	287.4034031	87.0752424
H3W12	H	12	34.7812528	45.43009408	53.618348	6.67085328	125.2075003	28.6041392
H4W12	H	12	102.9805977	110.1083817	94.36788349	0	194.8902876	29.59890946
H5W12	H	12	91.19115435	63.49150869	102.1191296	26.61763322	167.0440155	59.47098275
H6W12	H	12	88.44816469	74.24800406	92.83224844	14.986554	150.3807951	27.90338813
H7W12	H	12	97.58579388	72.09859505	169.900713	47.01018248	166.3485769	92.2491943
H8W12	H	12	193.8452448	158.5224219	358.7940711	187.8205536	484.3218951	166.8028485
H9W12	H	12	39.40141382	60.80953692	48.38186685	16.35150001	73.79295758	30.40480569
Mean HW12		12	104.3271342	91.74574016	139.8016117	46.88016716	206.1736789	65.2636888
Median HW12		12	94.38847411	73.17329956	98.24350655	21.48456661	166.6962962	44.93789422
H2W16	H	16	345.7682685	352.9520037	229.9578685	101.4742244	545.7348792	110.5816078
H3W16	H	16	121.961944	89.65175803	140.5605812	34.57684425	223.6253866	55.01264579
H4W16	H	16	356.4755102	177.0378146	200.5694668	0	406.3242621	49.64953397
H5W16	H	16	296.8275657	115.3765807	246.0181785	52.31238853	216.0489329	106.6407974
H6W16	H	16	478.4295847	240.0235721	385.9712519	28.40444228	610.3306249	95.78728912
H7W16	H	16	238.5105008	70.26186225	183.4327794	92.33571075	185.7028543	113.2950846
H8W16	H	16	50.74442078	98.94554797	72.7414275	71.75050281	95.35756984	72.26861438
H9W16	H	16	264.0959005	183.7812376	230.4884977	74.69194584	234.9172579	133.6396682
Mean HW16		16	269.1017119	166.0037971	211.2175064	56.94325736	314.755221	92.10940516
Median HW16		16	280.4617331	146.2071976	215.2636676	62.03144567	229.2713223	101.2140433
H2W18	H	18	452.9165137	300.1827442	233.9870369	45.57052849	370.6767452	72.749376
H3W18	H	18	661.9366492	222.5757718	461.3325974	83.76839775	432.4704099	157.9676132
H4W18	H	18	1289.905455	355.7164755	656.3448286	49.32793016	898.2295935	205.7054952
H5W18	H	18	1247.414948	516.9411764	898.4835551	277.8705386	606.4699932	331.7984207
H6W18	H	18	540.4257914	243.9880663	304.8942707	36.4140601	424.0521706	78.7722859
H7W18	H	18	696.5217883	211.5899305	584.6350686	316.09188	693.7976429	308.0960116
H8W18	H	18	63.0353352	283.3445073	296.9491749	119.364555	283.974789	227.6028339
H9W18	H	18	287.995701	106.8340587	181.4721066	36.8064849	188.5971126	118.6674504
Mean HW18		18	655.0190227	280.1465913	452.2623298	120.6517969	487.2835571	187.6699359
Median HW18		18	601.1812203	263.6662868	383.1134341	66.54816396	428.2612902	181.8365542
t-test HW12xW16			0.009513756	0.022993739	0.135956076	0.307198506	0.124671369	0.112338698
t-test Hw12xW18			0.005337088	0.001555427	0.008862019	0.06753788	0.013210789	0.004078454
t-test HW16xW18								

original Spot#	H or L	week	51_ALD 74	53_ABCA1 49	54_V-ATPase 13	55_SMT1 21	56_GAPDH 26	57_lipocalin 53
H2W12	H	12	58.68839938	93.05518978	247.9679754	83.54515675	166.7209481	334.3940602
H3W12	H	12	20.59257872	22.32321224	81.16033856	64.83957424	40.4361188	147.9456479
H4W12	H	12	65.13398483	90.64652135	324.8504275	171.1918429	94.66482511	305.2922037
H5W12	H	12	44.63786932	50.09171939	190.1616947	80.50924058	82.6706647	175.3464679
H6W12	H	12	26.78588175	50.4166995	195.6110681	137.3619381	104.2337002	164.7527733
H7W12	H	12	68.78978858	65.26115993	293.788349	140.4017245	196.9495448	184.0453814
H8W12	H	12	132.6655326	196.7939103	482.473662	380.5472436	376.5408089	490.8516224
H9W12	H	12	25.7024038	33.02118863	69.62464206	39.41220871	78.8243802	156.4980626
Mean HW12		12	55.37455487	75.20120014	235.7047697	137.2261162	142.6301238	244.8907774
Median HW12		12	51.66313435	57.83892971	221.7895218	110.4535474	99.44926263	179.6959247
H2W16	H	16	220.0007821	293.3707067	323.9250519	179.0566214	101.5211664	421.0777045
H3W16	H	16	82.75657021	30.22282853	174.7539858	207.4224283	251.1901827	309.6608579
H4W16	H	16	197.7346008	166.8977766	413.1754026	199.2266762	265.0093873	439.1006298
H5W16	H	16	190.3857164	115.4912458	202.7245298	188.9004686	142.0963089	329.6837561
H6W16	H	16	218.6304148	249.806371	609.4376009	382.2641558	339.5184806	522.8521218
H7W16	H	16	118.9975569	90.87733725	322.5331003	168.207513	175.5208	196.8973604
H8W16	H	16	69.23182422	89.8679975	110.6304081	81.13146734	46.57846023	200.9714324
H9W16	H	16	193.126268	122.9746672	437.5839066	176.880298	250.8472315	504.8360739
Mean HW16		16	161.3579667	144.9386163	324.3454982	197.8862036	196.5352522	365.6349921
Median HW16		16	191.7559922	119.2329565	323.2290761	183.978545	213.1840158	375.3807303
H2W18	H	18	299.1531655	198.1080803	334.3403986	163.0462461	134.2222153	415.6210688
H3W18	H	18	479.4000229	95.64974509	560.8643067	435.9271885	319.2086542	681.7140834
H4W18	H	18	799.6133038	371.7332005	1257.227272	789.7965928	324.7169406	954.2869278
H5W18	H	18	1047.558666	435.1928835	976.6173528	766.7318222	534.1618356	1178.670105
H6W18	H	18	302.5707693	230.6908032	550.1867542	348.6611596	238.1214133	447.5581433
H7W18	H	18	469.8149495	219.9572005	808.896297	487.5871065	502.0533411	437.7159537
H8W18	H	18	407.1345969	190.5590058	594.2814065	96.3212718	229.6302017	687.2214972
H9W18	H	18	274.4139885	41.95767405	335.5002609	174.964155	270.6918509	363.1911935
Mean HW18		18	509.9574329	222.9810741	677.2392561	407.8794428	319.1008066	645.7473716
Median HW18		18	438.4747732	209.0326404	577.5728566	392.294174	294.9502525	564.6361134
t-test HW12xW16			0.004743139	0.045795542	0.166062909	0.161219967	0.224144496	0.069361311
t-test Hw12xW18			0.001167062	0.008411331	0.002201901	0.021620038	0.01743159	0.003858552
t-test HW16xW18			0.004658255					

original Spot#	H or L	week	58_GPX 54	60_HSP17.6 80	63_ACTG2 68	68_PrxQ 51	75_HECT 30	76_FtsZ 32
H2W12	H	12	419.9827385	75.94197171	195.0746498	191.8985043	128.303738	166.5475289
H3W12	H	12	244.7814062	17.61356072	83.20785928	64.57827152	60.70783432	47.7215424
H4W12	H	12	336.1326832	25.03323764	124.3853555	112.6381258	26.17093364	84.42756096
H5W12	H	12	364.1518202	9.512590906	145.0647688	109.0113991	107.1576049	75.92472641
H6W12	H	12	326.2615091	23.98730466	176.8643648	126.052821	47.05119684	74.73518306
H7W12	H	12	316.8558248	33.60921541	199.0720671	178.5395426	57.74781709	142.0846025
H8W12	H	12	825.4727023	102.9660653	444.4388975	387.7576935	270.2307833	314.0717634
H9W12	H	12	175.9784049	11.03412454	101.6657666	70.90312897	32.46912319	41.04134335
Mean HW12		12	376.2021362	37.46225886	183.7217162	155.1724358	91.22987891	118.3192814
Median HW12		12	331.1970961	24.51027115	160.9645668	119.3454734	59.2278257	80.17614368
H2W16	H	16	704.4914327	88.75921829	197.870842	220.4594102	210.1532269	205.9253231
H3W16	H	16	434.7976931	26.69821896	204.2935913	170.2917341	52.50873099	135.1484409
H4W16	H	16	677.2710128	31.68221346	315.0697118	199.9105548	65.39122478	202.4841724
H5W16	H	16	557.6206386	23.29807586	273.918601	214.9392523	93.75830826	212.2958222
H6W16	H	16	1018.833082	69.85209309	582.3561248	283.6457015	264.8805114	221.006347
H7W16	H	16	406.5333444	47.19144534	224.2214326	207.3118001	60.95847009	149.6452813
H8W16	H	16	288.9318785	45.55035664	132.5013912	97.22181422	96.29887992	82.95622516
H9W16	H	16	674.8851727	37.55759917	387.8327877	323.4108906	41.60947231	145.0109238
Mean HW16		16	595.4205318	46.3236526	289.7580603	214.6488947	110.6948531	169.309067
Median HW16		16	616.2529056	41.5539779	249.0700168	211.1255262	79.57476652	176.0647268
H2W18	H	18	613.8815449	55.50195953	166.9849202	206.3668125	157.974246	189.914303
H3W18	H	18	1160.908729	50.04840028	514.2502029	481.5719785	205.6824297	343.1686751
H4W18	H	18	1796.085192	51.69066781	867.8765562	636.9702195	244.4811829	647.4847629
H5W18	H	18	2049.004835	103.5684053	931.3168008	946.8603113	219.4298165	702.8104818
H6W18	H	18	910.5681889	50.90544924	338.8159672	218.3728605	149.7310998	146.2072928
H7W18	H	18	1180.548727	114.1233005	564.5026371	636.8514347	320.6667844	411.4686953
H8W18	H	18	894.6425217	91.97596185	588.1372731	468.8349018	155.8890341	283.2516156
H9W18	H	18	560.7849557	35.96868915	299.1316487	299.8248663	61.87299915	116.7747276
Mean HW18		18	1145.803087	69.22285421	533.8770008	486.9566731	189.4659491	355.1350693
Median HW18		18	1035.738459	53.59631367	539.37642	475.2034402	181.8283379	313.2101453
t-test HW12xW16			0.064666146	0.212707805	0.102335268	0.162208493	0.314584652	0.140397729
t-test Hw12xW18			0.00354839	0.028493454	0.005769169	0.006089964	0.0258647	0.015511279
t-test HW16xW18								

original<br>Spot#	H or L	week	77_NACA<br>33	78_GST phi<br>41	79_U2-SnRNP<br>48	80_PLD<br>63	81_RPL10<br>72	83_Catalase<br>9
H2W12	H	12	52.36894433	248.4398193	45.1754441	78.04094775	34.34090351	268.7701131
H3W12	H	12	38.00046096	134.8919628	17.50974736	68.86161632	23.54281048	20.65782488
H4W12	H	12	23.32973046	209.2459223	18.64947429	110.9706901	8.627157656	142.3299915
H5W12	H	12	48.44025187	178.7771044	22.51250374	103.4320237	14.20750368	176.2002525
H6W12	H	12	30.94074356	176.9151127	28.56561413	117.4627322	24.18792328	212.0619931
H7W12	H	12	39.32074403	132.9422528	36.33992175	142.7134254	15.15842506	261.0885062
H8W12	H	12	74.415105	281.7699698	94.0406574	276.2072739	14.70670515	513.1701131
H9W12	H	12	12.65957403	111.7323342	12.97605036	33.32687006	8.784469986	23.19192242
Mean HW12		12	39.93444428	184.3393098	34.47117664	116.3769474	17.94448735	202.1838396
Median HW12		12	38.66060249	177.8461085	25.53905893	107.2013569	14.93256511	194.1311228
H2W16	H	16	99.90496085	391.5711896	60.86890893	325.5571206	45.05767734	155.6390693
H3W16	H	16	66.96585705	283.2002405	32.71829144	129.3490662	29.02300548	122.0910509
H4W16	H	16	84.36570259	382.9227193	55.82940785	204.4117474	66.88973481	243.7409434
H5W16	H	16	86.3929247	175.7835244	45.5351558	136.8488907	26.76859591	330.3777869
H6W16	H	16	133.1139481	511.1897865	95.24763751	269.7930155	66.72581142	710.4401851
H7W16	H	16	39.44159531	185.0339718	32.42442506	128.1478489	24.55726191	314.1117578
H8W16	H	16	39.20810531	207.0035091	20.69403563	58.81446563	13.56145477	166.1127463
H9W16	H	16	70.30012399	386.0573589	22.57513403	129.0064001	35.59159131	72.93515938
Mean HW16		16	77.46165224	315.3452875	45.73662453	172.7410694	38.52189162	264.4310874
Median HW16		16	77.33291329	333.0614799	39.12672362	133.0989784	32.30729839	204.9268449
H2W18	H	18	97.12253701	438.392237	77.77209613	255.6255557	52.90331646	183.0285303
H3W18	H	18	156.6612194	545.864324	77.25891113	301.3540493	82.65325397	519.409243
H4W18	H	18	279.2768194	924.2951056	159.3876024	516.3575027	139.2255923	736.5038514
H5W18	H	18	206.8075368	953.2922445	222.818115	408.3595783	100.7323562	1405.585531
H6W18	H	18	139.0358197	266.6501309	59.6017873	228.717654	53.68972826	578.3066827
H7W18	H	18	168.088843	556.065509	159.1566197	336.6506702	22.87259942	747.2754604
H8W18	H	18	119.5186068	432.3349892	49.424526	234.1733223	22.07303835	254.9420384
H9W18	H	18	90.7209087	255.5745464	31.0342791	141.7434183	25.73005905	82.98386715
Mean HW18		18	157.1540364	546.5586358	104.5567421	302.8727188	62.484993	563.5044005
Median HW18		18	147.8485196	492.1282805	77.51550363	278.4898025	53.29652236	548.8579628
t-test HW12xW16			0.018676034	0.01505067	0.226971377	0.139483951	0.012414965	0.242748337
t-test Hw12xW18			0.000962523	0.003166562	0.02017184	0.002956279	0.012536157	0.032083397
t-test HW16xW18								

original Spot#	H or L	week	83_Catalase 9	84_RbcL 56	85_MCCC1 67
H2W12	H	12	268.7701131	78.64589148	0.26177065
H3W12	H	12	20.65782488	46.09663072	2.82152864
H4W12	H	12	142.3299915	90.26728935	0
H5W12	H	12	176.2002525	105.8013475	28.67401945
H6W12	H	12	212.0619931	86.95486088	22.15662675
H7W12	H	12	261.0885062	83.49870458	9.210003425
H8W12	H	12	513.1701131	269.8236585	17.2724091
H9W12	H	12	23.19192242	37.43056516	0
Mean HW12		12	202.1838396	99.81486852	10.04954475
Median HW12		12	194.1311228	85.22678273	6.015766033
H2W16	H	16	155.6390693	216.1014718	0
H3W16	H	16	122.0910509	81.11086698	25.70667456
H4W16	H	16	243.7409434	170.6528029	19.80159254
H5W16	H	16	330.3777869	110.8185261	25.63686082
H6W16	H	16	710.4401851	301.356879	102.2163643
H7W16	H	16	314.1117578	87.51519581	0
H8W16	H	16	166.1127463	85.64413188	0
H9W16	H	16	72.93515938	72.92582859	0
Mean HW16		16	264.4310874	140.7657129	21.67018652
Median HW16		16	204.9268449	99.16686094	9.900796269
H2W18	H	18	183.0285303	196.9639286	14.87780672
H3W18	H	18	519.409243	225.3717583	58.847266
H4W18	H	18	736.5038514	415.0161906	110.7837068
H5W18	H	18	1405.585531	286.9133979	26.28105594
H6W18	H	18	578.3066827	217.0362024	35.271654
H7W18	H	18	747.2754604	271.5233736	0
H8W18	H	18	254.9420384	157.823766	0
H9W18	H	18	82.98386715	64.7104104	0
Mean HW18		18	563.5044005	229.4198785	30.75768619
Median HW18		18	548.8579628	221.2039803	20.57943133
t-test HW12xW16			0.242748337	0.175844409	0.160610528
t-test Hw12xW18			0.032083397	0.012295577	0.106194493
t-test HW16xW18					
